# Antipurinergic therapy corrects the autism-like features in the Fragile X (*Fmr1* knockout) mouse model

**DOI:** 10.1186/2040-2392-6-1

**Published:** 2015-01-13

**Authors:** Jane C Naviaux, Lin Wang, Kefeng Li, A Taylor Bright, William A Alaynick, Kenneth R Williams, Susan B Powell, Robert K Naviaux

**Affiliations:** The Mitochondrial and Metabolic Disease Center, University of California, San Diego School of Medicine, 214 Dickinson St., Bldg CTF, Rm C102, San Diego, CA 92103-8467 USA; Department of Medicine, University of California, San Diego School of Medicine, 214 Dickinson St., Bldg CTF, Rm C102, San Diego, CA 92103-8467 USA; Department of Pediatrics, University of California, San Diego School of Medicine, 214 Dickinson St., Bldg CTF, Rm C102, San Diego, CA 92103-8467 USA; Department of Pathology, University of California, San Diego School of Medicine, 214 Dickinson St., Bldg CTF, Rm C102, San Diego, CA 92103-8467 USA; Department of Psychiatry, University of California, San Diego School of Medicine, 214 Dickinson St., Bldg CTF, Rm C102, San Diego, CA 92103-8467 USA; Research Service, VA San Diego Healthcare System, La Jolla, CA USA; Veterans Affairs Center for Excellence in Stress and Mental Health (CESAMH), La Jolla, CA USA; General Atomics, Inc, San Diego, CA USA

**Keywords:** Autism spectrum disorders, Purinergic signaling, Antipurinergic therapy (APT), Mitochondria, Metabolism, Metabolomics, Fragile X syndrome, Genetics, Environment, Maternal immune activation (MIA), Cell danger response (CDR)

## Abstract

**Background:**

This study was designed to test a new approach to drug treatment of autism spectrum disorders (ASDs) in the Fragile X (*Fmr1*) knockout mouse model.

**Methods:**

We used behavioral analysis, mass spectrometry, metabolomics, electron microscopy, and western analysis to test the hypothesis that the disturbances in social behavior, novelty preference, metabolism, and synapse structure are treatable with antipurinergic therapy (APT).

**Results:**

Weekly treatment with the purinergic antagonist suramin (20 mg/kg intraperitoneally), started at 9 weeks of age, restored normal social behavior, and improved metabolism, and brain synaptosomal structure. Abnormalities in synaptosomal glutamate, endocannabinoid, purinergic, and IP3 receptor expression, complement C1q, TDP43, and amyloid β precursor protein (APP) were corrected. Comprehensive metabolomic analysis identified 20 biochemical pathways associated with symptom improvements. Seventeen pathways were shared with human ASD, and 11 were shared with the maternal immune activation (MIA) model of ASD. These metabolic pathways were previously identified as functionally related mediators of the evolutionarily conserved cell danger response (CDR).

**Conclusions:**

The data show that antipurinergic therapy improves the multisystem, ASD-like features of both the environmental MIA, and the genetic Fragile X models. These abnormalities appeared to be traceable to mitochondria and regulated by purinergic signaling.

**Electronic supplementary material:**

The online version of this article (doi:10.1186/2040-2392-6-1) contains supplementary material, which is available to authorized users.

## Background

Autism spectrum disorders (ASDs) now affect 1% to 2% of children in the United States [1]. Genetic [2–4], environmental [5, 6], and metabolic [7] factors can contribute to the risk of ASD to different extents in each affected child. We have previously shown that antipurinergic therapy reverses the behavioral and metabolic abnormalities in the maternal immune activation (MIA) mouse model of ASD in juveniles [8] and adults [9]. The MIA and Fragile X models have been considered to be mechanistically distinct examples of environmental and genetic causes of ASD, respectively. However, in our MIA study we found the first of several emerging connections. The *Fmr1* protein (FMRP) was downregulated by 50%, and antipurinergic therapy with suramin restored normal FMRP and normal behaviors in the MIA model [8]. FMRP is an mRNA and ribosome [10] binding protein that inhibits the expression of several key inflammatory proteins and cytokines, and binds to several DNA repair proteins involved in cell stress and defense [11]. Genetic loss of FMRP expression leads to Fragile X Syndrome, the most common single-gene cause of intellectual disability [12]. The *Fmr1* knockout is the oldest, and one of the most studied genetic mouse models used in autism research [13]. In our previous work we found that disturbances in purine metabolism and purinergic signaling were robust features and effective targets for treatment in the environmental MIA mouse model of autism [8, 9]. Interestingly, the first genetic causes of autism identified were traced to abnormalities in purine and pyrimidine metabolism [14, 15]. These observations led us to test the role of purinergic signaling in a genetic mouse model of ASD. We selected the Fragile X model to test the hypothesis that abnormalities in purinergic signaling might underlie both the environmental MIA and genetic Fragile X models.

Suramin is a well-known and well-studied competitive inhibitor of purinergic signaling [16]. It has been used medically for the treatment of African sleeping sickness (trypanosomiasis) since shortly after it was first synthesized in 1916. Its antipurinergic actions were discovered in 1988, after a search for inhibitors of ATP-mediated P2X and P2Y signaling [17]. Suramin has many other actions [18], however, metabolomic studies have shown that the expression of purinergic receptors is altered [8], and purine metabolism is the top ranked biochemical pathway that is changed by treatment in the MIA model of ASD [9]. We refer to the use of suramin and related purinergic antagonists as antipurinergic therapy (APT). In the present work, we tested the hypothesis that APT will improve behavior, metabolism, and synaptic abnormalities in the Fragile X mouse model, even in the face of a permanent, gene-coded absence of the Fragile X protein.

## Methods

### Mouse strains

We evaluated the Fragile X (*Fmr1*) knockout on the FVB strain background. It has the genotype: FVB.129P2-*Pde6b*^+^*Tyr*^c-ch^*Fmr1*^tm1Cgr^/J (Jackson Stock # 004624). The *Fmr1*^tm1Cgr^ allele contains a neomycin resistance cassette replacing exon 5 that results in a null allele that makes no FMR mRNA or protein. The control strain used has the genotype: FVB.129P2-*Pde6b*^+^*Tyr*^c-ch^/AntJ (Jackson Stock # 004828). In contrast to the white coat color of wild-type FVB mice, these animals had a chinchilla (*Tyr*^c-ch^) gray coat color. The wild-type *Pde6b* locus from the 129P2 ES cells corrects the retinal degeneration phenotype that produces blindness by 5 weeks of age in typical FVB mice. The *Fmr1* locus is X-linked, so males are hemizygous and females are homozygous for the knockout. We also performed metabolomic analysis on *Fmr1* knockout mice on the C57BL/6J (B6) background to refine our understanding of which metabolic disturbances were directly related to the *Fmr1* knockout, and which were the result of changes in genetic background. For these studies we studied the same *Fmr1*^tm1Cgr^ knockout allele bred on the C57BL6/J background. These animals had the genotype: B6.129P2-*Fmr1*^*tm1Cgr*^/J (Jackson Stock# 003025). The standard C57BL6/J strain (Jackson Stock# 000664) was used as a control for the B6 metabolic studies.

### Animals, husbandry, and drug treatment

All studies were conducted at the University of California, San Diego (UCSD) in facilities accredited by the Association for Assessment and Accreditation of Laboratory Animal Care International (AAALAC) under UCSD Institutional Animal Care and Use Committee (IACUC)-approved animal subjects protocols, and followed the National Institutes of Health (NIH) Guidelines for the use of animals in research. Five-week-old male mice were obtained from Jackson Laboratories (Bar Harbor, ME), identified by ear tags, placed in cages of two to four animals, and maintained on *ad libitum* Harlan Teklad 8604 mouse chow (14% fat, 54% carbohydrate, 32% protein) and water. Animals were housed in a temperature (22°C to 24°C) and humidity (40% to 55%) controlled vivarium with a 12-h light-dark cycle (lights on at 07:00). No mice were housed in isolation. Beginning at 9 weeks of age, animals received weekly injections of either saline (5 μL/g ip) or suramin (hexasodium salt, 20 mg/kg ip; Tocris Cat #1472).

### Behavioral analysis

Behavioral testing began at 13 weeks of age, after 1 month of weekly antipurinergic therapy with suramin. Mice were tested in social approach, T-maze, locomomtor activity, marble burying, acoustic startle, and prepulse inhibition paradigms as follows. The ages at the time of testing are noted in the figure legends. For a complete description of the behavioral paradigms see Full Methods Online. *Social Preference and Social Novelty*. Social behavior was tested as social preference as previously described [9], with the addition of a third phase with a second novel mouse to interrogate social novelty [19]. *T-Maze*. Novelty preference was tested as spontaneous alternation behavior in the T-maze as previously described [9]. *Marble Burying*. Marble burying behavior was measured over 30 min by a modification of methods used by Thomas *et al.*[20]. *Locomotor Activity*. Locomotor activity, hyperactivity (total distance traveled), center entries, holepoke exploration, and vertical investigation (rearing) behaviors were quantified by automated beam break analysis in the mouse behavioral pattern monitor (mBPM) as previously described [21]. *Acoustic Startle and Prepulse Inhibition*. Sensitivity to acoustic startle and prepulse inhibition of the startle reflex were measured by automated testing in commercial startle chambers as previously described [22].

### Body temperature measurements

A BAT-12 Microprobe digital thermometer and RET-3 mouse rectal probe (Physitemp Instruments, Clifton, NJ, USA) were used to obtain rectal core temperatures to a precision of +/-0.1°C, as previously described [8]. Care was taken to measure temperatures ≥2 days after cage bedding changes, and to avoid animal transport stress immediately prior to measurement in order to avoid stress-induced hyperthermia [23]. Temperatures were measured between 09:00 and 12:00 each day.

### Synaptosome isolation and ultrastructure

Animals were sacrificed at 25 weeks of age, after receiving 16 weeks of treatment with suramin or saline. Cerebral samples were collected, homogenized, and synaptosomes isolated by discontinuous Percoll gradient centrifugation, drop dialyzed, glutaraldehyde fixed, post-fixed in osmium tetroxide, embedded, sectioned, and stained with uranyl acetate for transmission electron microscopy (TEM) as previously described [8]. Samples from the FVB control animals (+/- suramin) were not available for study by either electron microscopy or western analysis. Therefore, we report only the effects of suramin on the two groups of *Fmr1* knockout animals (KO-saline and KO-suramin). N = 3 animals/group. Four to six TEM images were collected from each sample. One 5,800× survey image, and three to five images of informative fields at 34,000× to 64,000× were collected with internal scale bars for dimensional control. Qualitatively representative images were reported.

### Western blot analysis

Twenty micrograms of cerebral synaptosomal protein was loaded in SDS-polyacrylamide gels (NuPage 4-12% gradient, Bis-Tris Gels) and transferred to PVDF membranes as previously described [8]. The blots were first stained with 0.1% Ponceau S in 5% acetic acid for 10 min, washed, scanned, and the transfer efficiency was quantified by densitometry. Blots were then blocked with 5% skim milk in tris-buffered saline with 0.1% Tween 20 (TBST) for 1 h at room temperature with shaking. Primary antibodies were obtained commercially as 1 mg/mL stocks (see Additional file 1: Table S1 for source details). These were diluted 1:500 to 1:10,000 (final concentrations of 2000 ng/mL to 100 ng/mL; Additional file 1: Table S1) in 5% BSA or 5% skim milk in TBS with 0.1% Tween 20 and optimized for each target to achieve signals in the linear range using dilutions (5 to 20 μg/lane) of cerebral synaptosomes prepared from C57BL/6J control animals. When monoclonal antibodies to a peptide of the target protein were used, peptide was pre-incubated with primary antibody to confirm specificity. When blocking peptides were not available, signal specificity was determined by correspondence of the observed band pattern and molecular weight to the published or manufacturer values for each target protein. Only antibodies that identified specific target bands in cerebral synaptosomes from age-matched control animals were used. Blots were probed with the optimized dilution of primary antibody overnight in the cold room (4°C). Secondary antibodies conjugated to horseradish peroxidase were obtained from Pierce (Rockford, IL, USA) diluted 1:5,000 (200 ng/mL) to 1:20,000 (50 ng/mL) in 3% skim milk-TBST. The blots were probed for 1 h at room temperature prior to final wash and signal development by enhanced chemiluminescence (ECL) or SuperSignal West Femto chemiluminsescent substrate (Thermo, Cat# 34095), then quantified by densitometry. At least five animals per group were analyzed. We evaluated the cerebral synaptosome expression of 54 proteins (Additional file 1: Table S1).

### Metabolomics

Broad spectrum analysis of 673 targeted metabolites from 60 biochemical pathways was performed as described [9], with minor modifications. Samples were analyzed on an AB SCIEX QTRAP 5500 triple quadrupole mass spectrometer equipped with a Turbo V electrospray ionization (ESI) source, Shimadzu LC-20A UHPLC system, and a PAL CTC autosampler (AB ACIEX, Framingham, MA, USA). Whole blood was collected 3 to 4 days after the last weekly dose of suramin (20 mg/kg ip) or saline (5 μL/g ip), after light anesthesia in an isoflurane (Med-Vet International, Mettawa, IL, USA, Cat# RXISO-250) drop jar, into BD Microtainer tubes containing lithium heparin (Becton Dickinson, San Diego, CA, USA, Ref# 365971) by submandibular vein lancet [24]. Plasma was separated by centrifugation at 600g × 5 min at 20°C within 1 h of collection. Fresh lithium-heparin plasma was transferred to labeled tubes for storage at -80°C for analysis. Typically 45 μL of plasma was thawed on ice and transferred to a 1.7 mL Eppendorf tube. A total of 2.5 μL of a cocktail containing 35 commercial stable isotope internal standards, and 2.5 μL of 310 stable isotope internal standards that were custom-synthesized in *E. coli* and *S. cerevisiae* by metabolic labeling with ^13^C-glucose and ^13^C-bicarbonate, were added, mixed, and incubated for 10 min at room temperature to permit small molecules and vitamins in the internal standards to associate with plasma binding proteins. Macromolecules (protein, DNA, RNA, and so on) were precipitated by extraction with 4 volumes (200 μL) of cold (-20°C), acetonitrile:methanol (50:50) (LCMS grade, Cat# LC015-2.5 and GC230-4, Burdick & Jackson, Honeywell), vortexed vigorously, and incubated on crushed ice for 10 min, then removed by centrifugation at 16,000 g × 10 min at 4°C. The supernatants containing the extracted metabolites and internal standards in the resulting 40:40:20 solvent mix of acetonitrile:methanol:water were transferred to labeled cryotubes and stored at -80°C for LC-MS/MS (liquid chromatography-tandem mass spectrometry) analysis.

LC-MS/MS analysis was performed by multiple reaction monitoring (MRM) under Analyst v1.6.1 (AB SCIEX, Framingham, MA, USA) software control in both negative and positive mode with rapid polarity switching (50 ms). Of the 673 metabolites targeted, 477 metabolites were measured by scheduled MRM in the first injection, and 196 metabolites were measured by scanning MRM in a second injection. Nitrogen was used for curtain gas (set to 30), collision gas (set to high), ion source gas 1 and 2 (set to 35). The source temperature was 500°C. Spray voltage was set to -4,500 V in negative mode and 5,500 V in positive mode. The values for Q1 and Q3 mass-to-charge ratios (m/*z*), declustering potential (DP), entrance potential (EP), collision energy (CE), and collision cell exit potential (CXP) were determined and optimized for each MRM for each metabolite. Ten microliters of extract were injected by PAL CTC autosampler into a 250 × 2 mm, 5 μm Luna NH2 aminopropyl HPLC column (Phenomenex, Torrance, CA, USA) held at 25°C for chromatographic separation. The mobile phase was solvent A: 95% water with 23.18 mM NH_4_OH (Sigma-Aldrich, St. Louis, MO, USA, Fluka Cat# 17837-100ML), 20 mM formic acid (Sigma, Fluka Cat# 09676-100ML) and 5% acetonitrile (pH 9.44); solvent B: 100% acetonitrile. Separation was achieved using the following gradient: 0 min 95% B, 3 min 95% B, 3.1 min 80% B, 6 min 80% B, 6.1 min 70% B, 10 min 70% B, 18 min 2% B, 27 min 0% B, 32 min 0% B, 33 min 100% B, 36.1 95% B, 40 min 95% B end. The flow rate was 300 μL/min. All the samples were kept at 4°C during analysis. The chromatographic peaks were identified using MultiQuant (v3.0, AB SCIEX), confirmed by manual inspection, and the peak areas integrated. The median of the peak area of stable isotope internal standards was calculated and used for the normalization of metabolites concentration across the samples and batches. Prior to multivariate and univariate analysis, the data were log-transformed.

### Metabolic pathway visualization in cytoscape

We constructed a rendering of mammalian intermediary metabolism in Cytoscape v 3.1.1 (http://www.cytoscape.org/). Pathways represented in the network for Fragile X syndrome included the 20 metabolic pathways and the 58 metabolites that were altered by antipurinergic therapy with suramin (VIP scores > 1.5). Nodes in the Cytoscape network represent metabolites within the pathways and have been colored according to the z-score. The z-score was computed as the arithmetic difference between the mean concentration of each metabolite in the KO-Sur treatment group and the KO-Sal control group, divided by the standard deviation in the controls. Node colors were arranged on a red-green color scale with green representing ≤-2.00 z-score, red representing ≥+2.00 z-score, and with a zero (0) z-score represented as white. The sum of the VIP scores of those metabolites with VIP scores >1.5 for each metabolic pathway is displayed next to the pathway name.

### Data analysis

Group means and standard error of the means (SEM) are reported. Behavioral data were analyzed by two-way ANOVA and one-way ANOVAs (GraphPad Prism 5.0d, GraphPad Software Inc., La Jolla, CA, USA, or Stata/SE v12.1, StataCorp, College Station, TX, USA). Pair-wise post hoc testing was performed by the method of Tukey or Newman-Keuls. Significance was set at *P* <0.05. Metabolomic data were log-transformed and analyzed by multivariate partial least squares discriminant analysis (PLSDA) in MetaboAnalyst [25]. Metabolites with variable importance in projection (VIP) scores greater than 1.5 were considered significant.

## Results

### Confirmation of Fragile X protein knockout

We confirmed the absence of Fragile X protein (FMRP) expression in *Fmr1* knockout mice, and its presence in FVB and C57BL/6J controls by western blot analysis before phenotyping the *Fmr1* knockout animals used in this study (Additional file 1: Figure S1).

### Restoration of normal social behavior

**Figure 1:**
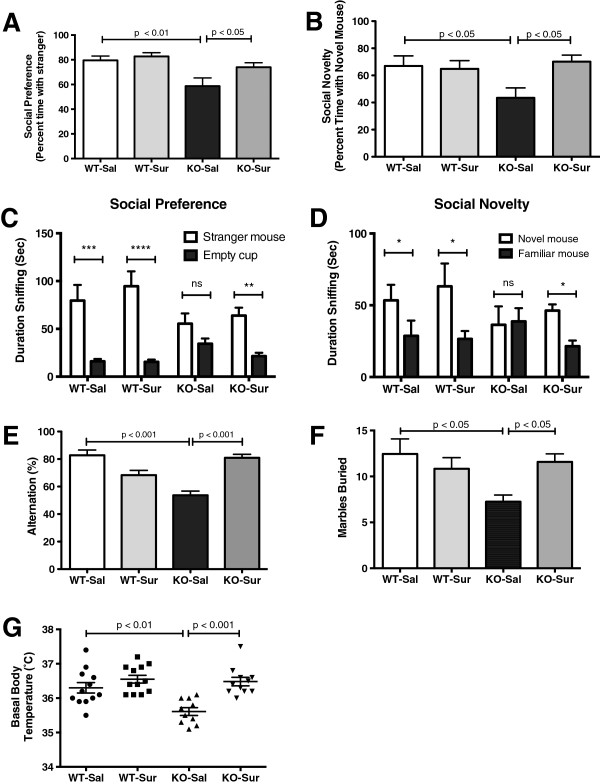
**ASD-like symptoms were improved by antipurinergic therapy. (A)** Social preference measured as percent time. *Fmr1* knockouts treated with saline showed significant deficits in social preference compared to wild-type controls (F(3,38) = 5.94, *P* = 0.002) and suramin corrected this (*P* <0.05). **(B)** Social novelty measured as percent time. *Fmr1* knockouts treated with saline also showed significant deficits in social novelty. (F(3,38) = 3.49, *P* = 0.025) and suramin restored this (*P* <0.05). **(C)** Social preference as absolute time spent interacting socially. *Fmr1* knockouts were less social than wild-type controls and suramin treatment corrected this (cup F(1,76) = 56.5, *P* = 0.0001; cup x group F(3,76) = 3.55, *P* = 0.018). **(D)** Social novelty as absolute time interacting with a novel mouse. *Fmr1* knockouts showed decreased novelty preference and suramin improved this (social stimulus main effect F(1,76) = 8.6; *P* = 0.004); social stimulus x group F(3,76) = 3.1, *P* = 0.032. Age 17 weeks; N = 9-12 per group for Social Preference/Social Novelty test. **(E)** Restoration of spontaneous alternation in the T-maze. Suramin improved spontaneous alternation in the *Fmr1* knockouts, but had not effect on FVB controls (F(3,41) = 16.6; *P* <0.0001). Age 13 weeks; N = 11-12 per group. **(F)** Restoration of normal marble burying. *Fmr1* knockouts treated with saline buried fewer marbles compared to controls (F(3,37) = 3.15; *P* = 0.037) and suramin corrected this (*P* <0.05). Age 16 weeks; N = 9-12 per group. **(G)** Relative hypothermia in the Fragile X model and correction with suramin. *Fmr1* knockout animals treated with saline had core temperatures that were 0.5-0.7°C lower than wild-type FVB controls (F(3,41) = 10.45, *P* <0.0001). Suramin restored normal body temperature in *Fmr1* knockouts (*P* = 0.001). Age 15 weeks, N = 11-12 per group. Values are expressed as means +/- SEM.

Altered social behavior is a key measure of autism-like features in mouse models of autism. In the Fragile X knockout genetic model of autism, it has also proven to be one of the most reproducible paradigms across different studies reported in the literature [26]. We found that *Fmr1* null males showed a 26% reduction in social preference, as measured by the time spent interacting with a stranger mouse compared to an inanimate object. There was also a 35% reduction in social novelty, as measured by the time spent interacting with a novel mouse compared to a familiar mouse. This altered social behavior was corrected by antipurinergic therapy with suramin (Figure 1A-D).

### Restoration of spontaneous alternation in the T-maze

Novelty preference is an innate feature of normal rodent [27] and human [28] behavior, and a predictor of socialization and communication growth in children with ASD [29]. The loss or suppression of novelty preference in children with ASD is associated with the phenomenon known as insistence on sameness [30]. We estimated preference for novelty as spontaneous alternation behavior in the T-maze [9]. The T-maze can also be used to estimate spatial working memory, especially when food motivated [31]. We did not use the food-motivated variation in our study. We found that the *Fmr1* null mice showed deficient novelty preference as reflected by chance (near 50%) spontaneous alternation behavior. These deficits were normalized by suramin treatment (Figure 1E). Fragile X knockout mice were no different from controls in latency to choice (data not shown).

### Restoration of marble burying behavior

We measured marble burying as a measure of normal rodent digging behavior. Marble burying has sometimes been considered a measure of anxiety, however, comprehensive genetic and behavioral studies have shown that marble burying is a normal mouse behavior that is genetically determined [20]. We found that marble burying was diminished 38% in Fragile X knockout mice. Suramin improved this (KO-Sal v KO-Sur; Figure 1F).

### Restoration of normal body temperature

*Fmr1* knockout mice displayed relative hypothermia of approximately 0.5°C to 0.7°C below the basal body temperature of the FVB controls (Figure 1G). This relative hypothermia was lost in stressed animals (data not shown). The maternal immune activation (MIA) mouse model showed a similar mild reduction in body temperature [8]. Normal basal body temperature was restored by antipurinergic therapy with suramin. Suramin had no effect on the body temperature of control animals (WT-Sal vs WT-Sur, Figure 1G).

### Synaptosomal ultrastructure and protein expression

**Figure 2:**
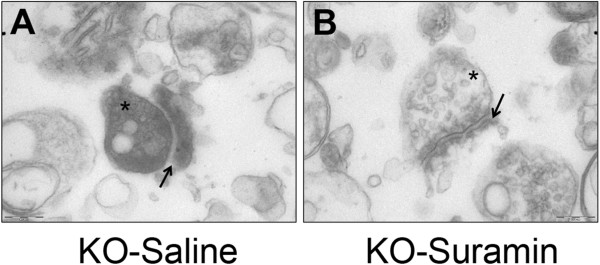
**Cerebral synaptosome structural abnormalities were improved by antipurinergic therapy. (A)** Fragile X knockout model treated with saline (KO-Saline). Note the increased electron density (dark staining) of the synaptosomal matrix (*) and the hypomorphic (thin) margins of the post-synaptic density (indicated by the arrow). Age 25 weeks, N = 3 per group. Scale bar = 200 nm. **(B)** Fragile X knockout model treated with suramin (KO-Suramin). Note the normal appearing, electron lucent synaptosomal matrix (*) and the thicker margins of the post-synaptic density (indicated by the arrow). Age 25 weeks, N = 3 per group. Scale bar = 200 nm.

Our previous studies showed synaptic ultrastructural abnormalities in the MIA mouse model that were corrected by antipurinergic therapy [8]. In that study, the animals with ASD-like behaviors were found to have abnormal synaptosomes containing an electron dense matrix and brittle or fragile and hypomorphic post-synaptic densities. In the present study of the Fragile X model, saline-treated *Fmr1* knockout mice had cerebral synaptosomes that also contained an electron dense matrix (Figure 2A, marked with an asterisk), and fragile, hypomorphic post-synaptic densities (Figure 2A, marked with an arrow). Normal appearing synaptosomes were also found in the *Fmr1* knockout animals, reflecting the well-known heterogeneity in synaptic maturation and morphology in this model. Suramin-treated mice had more cerebral synaptosomes that were near-normal in appearance, with an electron lucent matrix (Figure 2B, marked with an asterisk), and normal appearing post-synaptic densities (Figure 2B, marked with an arrow). We did not investigate dendritic spine densities in this study.

### Cerebral synaptosomal protein analysis

**Figure 3:**
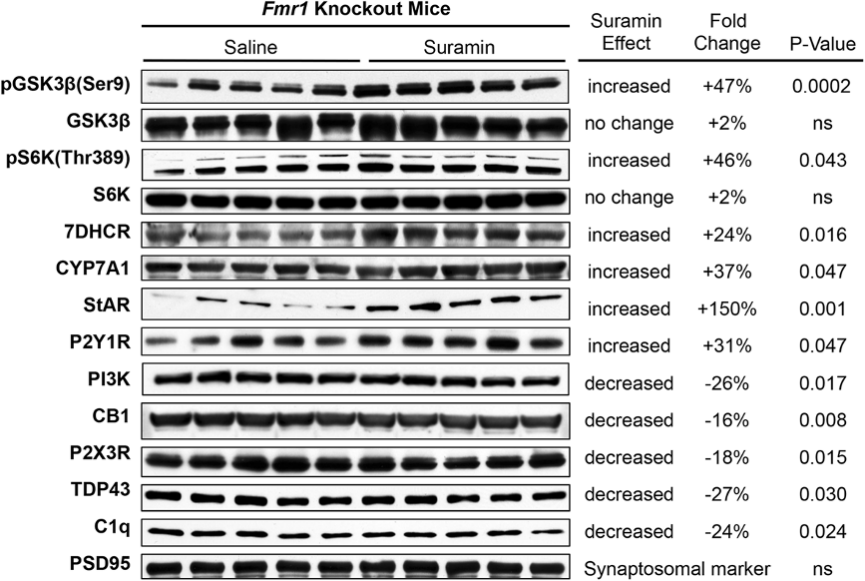
**Western analysis of cerebral synaptosomal proteins changed by suramin treatment.** Seventeen of 54 interrogated proteins were found to be changed by suramin treatment measured at 25 weeks of age. Post-synaptic density protein 95 kD (PSD95) was not influenced by suramin treatment and was used as a loading control. Protein expression data quantified by densitometry.

**Figure 4:**
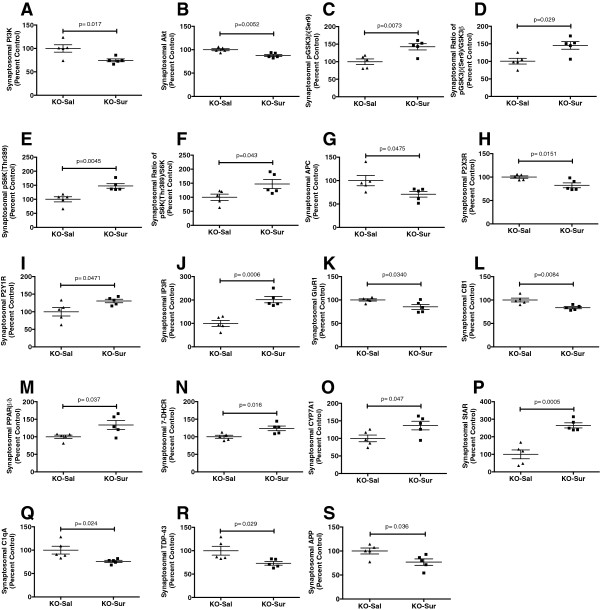
**Cerebral synaptosomal proteins changed by antipurinergic therapy with suramin. (A)** Phosphoinositol trisphosphate kinase (PI3K). **(B)** Akt (protein kinase B). **(C)** Serine 9 phosphorylation of glycogen synthase kinase (pGSK3β). **(D)** Phosphorylation ratio of glycogen synthase kinase 3β (pGSK3β/GSK3β). **(E)** Phosphorylated of ribosomal protein S6 Kinase (pS6K). **(F)** Phosphorylation ratio of pS6K/S6K. **(G)** Adenomatous polyposis coli (APC). **(H)** Purinergic receptor P2X3 (P2X3R). **(I)** Purinergic receptor P2Y1 (P2Y1R). **(J)** Inositol trisphosphate receptor (IP3R). **(K)** Glutamate receptor 1 (GluR1, also known as the AMPA receptor). **(L)** Cannabinoid receptor 1 (CB1). **(M)** Peroxisome proliferator activated receptor β (PPARβ, also known as PPARδ). **(N)** 7-Dehydrocholesterol dehydrogenase (7-DHCR). **(O)** Cholesterol 7α-hydroxylase (CYP7A1). **(P)** Steroidogenic acute response protein (StAR). **(Q)** Activated complement protein C1q (C1qA). **(R)** TAR DNA binding protein 43 (TDP43). **(S)** Amyloid-β precursor protein (APP). Age = 25 weeks, N = 5 per group. Values are expressed as means +/- SEM.

We found that 17 of 54 proteins we interrogated in cerebral synaptosomes (see Additional file 1: Table S1) were changed by antipurinergic therapy with suramin in the Fragile X model (Figures 3 and 4; KO-Sur vs. KO-Sal). As a treatment study, we focused on the effect of suramin in the *Fmr1* knockout mice only. The current study did not compare knockout brain protein levels to littermate FVB controls (see Methods).

### Synaptosomal PI3K/AKT/GSK3β pathway

The PI3/AKT/GSK3β pathway is pathologically elevated in the Fragile X model [32]. Suramin inhibited this pathway at several points. Suramin decreased the expression of PI3 Kinase and AKT, and increased the inhibitory phosphorylation of the PI3K/AKT pathway protein glycogen synthase kinase 3β (pGSK3β^Ser9^) by 47%. Suramin increased the phosphorylation of p70 S6 kinase (pS6K^Thr389^) by 46% (Figures 3 and 4A-F). Phosphorylated p70 S6K^Thr389^ is a negative regulator of insulin receptor substrate 1 (IRS1), and serves to oppose insulin stimulated cell growth, PI3K, and mTORC1 signaling [33]. We did not find a corresponding change in mTOR expression or phosphorylation in cerebral synaptosomes of the Fragile X model (Additional file 1: Table S1).

### Synaptosomal adenomatous polyposis coli (APC) expression

APC is a tumor suppressor protein that is increased in the Fragile X knockout model [34]. APC forms a complex with, and is phosphorylated by, active GSK3β to inhibit microtubule assembly during undifferentiated cell growth of neuronal progenitors [35]. Suramin treatment returned total APC protein to control levels by decreasing expression by 29% (Figure 4G).

### Synaptosomal purinergic receptors and the IP3R1 calcium channel

In earlier studies we showed the chronic hyperpurinergia associated with the MIA mouse model resulted in downregulated expression of the P2Y2 receptor. Suramin treatment in the MIA model increased P2Y2 expression to normal levels [8]. In the Fragile X mouse model, suramin treatment increased the expression of the P2Y1 receptor 32%, and decreased P2X3 receptor expression 18% (Figure 4HI). There was no effect on P2Y2 expression (Additional file 1: Table S1). P2Y1 signaling is known to inhibit IP3 gated calcium release from the endoplasmic reticulum [36]. We found that suramin treatment was associated with a 101% increase in IP3R1 expression (Figure 4J).

### Synaptosomal AMPA receptor (GluR1) expression

AMPA receptor (GluR1) mRNA transcription, translation, and receptor recycling are known to be pathologically dysregulated in the Fragile X model [37]. In the lateral amygdala, the Fragile X knockout results in enhanced internalization and increased internalized receptor pools, with decreased surface expression, such that the total mass of the AMPA receptor is unchanged from controls [38]. Suramin treatment decreased the overall expression of the ionotropic GluR1 in cerebral synaptosomes by 15% (Figure 4K). However, these methods were unable to distinguish between surface and internalized pools of AMPA receptors. Suramin had no effect on metabotropic glutamate receptor mGluR5 expression in this model (Additional file 1: Table S1).

### Synaptosomal cannabinoid receptor expression

Cannabinoid signaling is pathologically increased in the *Fmr1* knockout model [39]. Suramin treatment decreased brain CB1 receptor expression 16% (Figure 4L). This is consistent with recent data that have shown signaling to be sharply increased in response to brain injury [40]. Pharmacologic blockade with the CB1R antagonist rimonabant has been shown to improve several symptoms in the Fragile X model [41]. CB2 expression is increased in the peripheral blood monocytes of children with autism spectrum disorders [42]. However, CB2 receptor expression in the brain synaptosomes of the Fragile X model was unchanged (Additional file 1: Table S1).

### Synaptosomal PPARβ/δ expression

PPARβ (also known as PPARδ) is a widely expressed transcriptional co-activator that is correlated with the aerobic and bioenergetic capacity in a variety of tissue types [43]. Suramin treatment increased the expression of PPARβ/δ in purified brain synaptosomes by 34% (Figure 4M). Suramin treatment had no effect on synaptosomal PPARα (Additional file 1: Table S1).

### Synaptosomal cholesterol and bile acid regulatory proteins

Antipurinergic therapy with suramin increased three key proteins involved in sterol and bile acid synthesis. 7-dehydrocholesterol reductase (7DHCR) was increased by 24%, cholesterol 7α-hydroxylase (CYP7A1) by 37%, and the steroidogenic acute regulatory (StAR) protein by 150% (Figure 4N-P) above saline treated control levels. The function of bile salts in the brain is unknown, although their neuroprotective effects have been shown in several models [44, 45].

### Synaptosomal complement C1q and TDP43

Recent studies have revealed an important role for complement proteins in tagging synapses during inflammation and remodeling [46]. Activated complement proteins have also been found in the brains of children with autism [47]. We found that suramin decreased synaptosomal C1qA by 24% (Figure 4Q).

Tar-DNA binding protein 43 (TDP43) is a single-strand DNA and RNA binding protein that disturbs mitochondrial transport and function under conditions of cell stress [48]. Mutations in TDP43 are associated with genetic forms of amyotrophic lateral sclerosis (ALS) [49]. Wild-type TDP43 protein is a component of the tau and α-synuclein inclusion bodies found in Alzheimer’s and Parkinson’s disease and plays a role in RNA homeostasis and protein translation [50]. The similarities of these functions to the role of the *Fmr1* gene in RNA homeostasis prompted us to investigate TDP43 in the Fragile X model. We found that suramin treatment decreased synaptosomal TDP43 by 27% (Figure 4R).

### Synaptosomal amyloid-β precursor protein expression

Amyloid-β precursor protein (APP) expression is upregulated in the brain of subjects with ASD [51]. A number of recent papers have identified the upregulation of gene networks in ASD [51] and inborn errors of purine metabolism [52] that were formerly thought to be specific for Alzheimer’s and other neurodegenerative disorders. We found that antipurinergic therapy with suramin decreased synaptosomal APP levels by 23% in the Fragile X model (Figure 4S).

### Synaptosomal protein pertinent negatives

We interrogated the effect of suramin on several additional proteins that were found to be dysregulated in the MIA mouse model [8]. We found no effect of suramin in the Fragile X model on ERK 1 and 2, or its phosphorylation, CAMKII or its phosphorylation, nicotinic acetylcholine receptor alpha 7 subunit (nAchRα7) expression, or the expression of the purinergic receptors P2Y2 and P2X7 (Additional file 1: Table S1). These data show that the detailed molecular effects of antipurinergic therapy with suramin are different in different genetic backgrounds and different mechanistic models of autism spectrum disorders. However, the efficacy in restoring normal behavior and brain synaptic morphology cuts across models. These data support the novel conclusion that antipurinergic therapy is operating by a metabolic mechanism that is common to, and underlies, both the environmental MIA, and the genetic Fragile X models of ASD.

### Metabolomic response to suramin treatment

**Figure 5:**
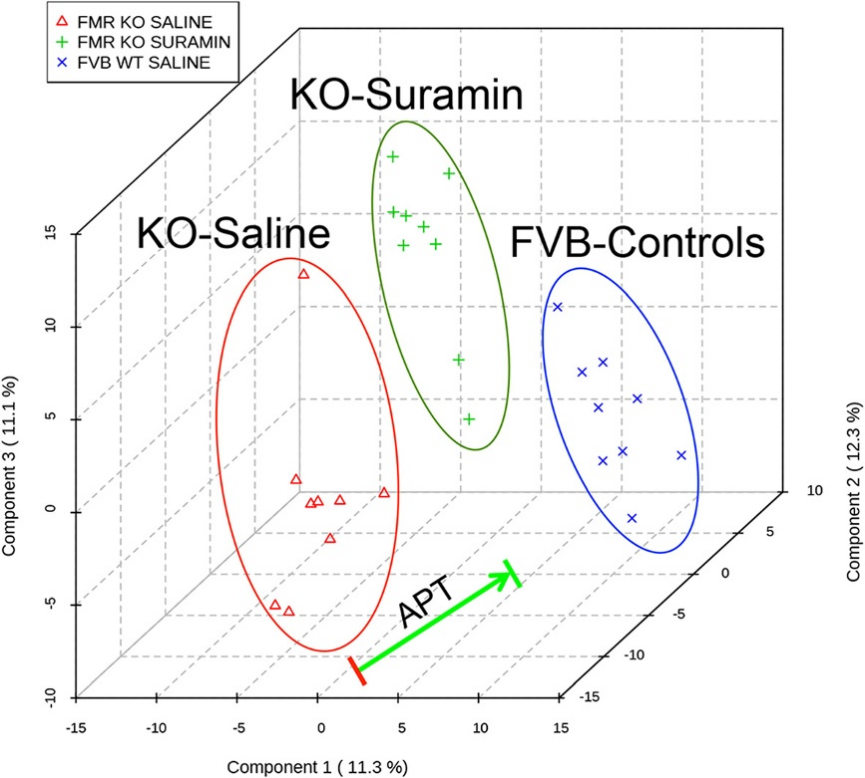
**Antipurinergic therapy improved the widespread metabolomic abnormalities in the Fragile X mouse model.** A total of 673 plasma metabolites from 60 biochemical pathways were measured by liquid chromatography tandem mass spectrometry (LC-MS/MS) and analyzed by partial least squares discriminant analysis (PLSDA). The three top multivariate components were then plotted on x, y, and z-axes, respectively. Suramin treatment shifted metabolism in the direction of wild-type controls. Age 25 weeks, N = 9-11 per group.

**Figure 6:**
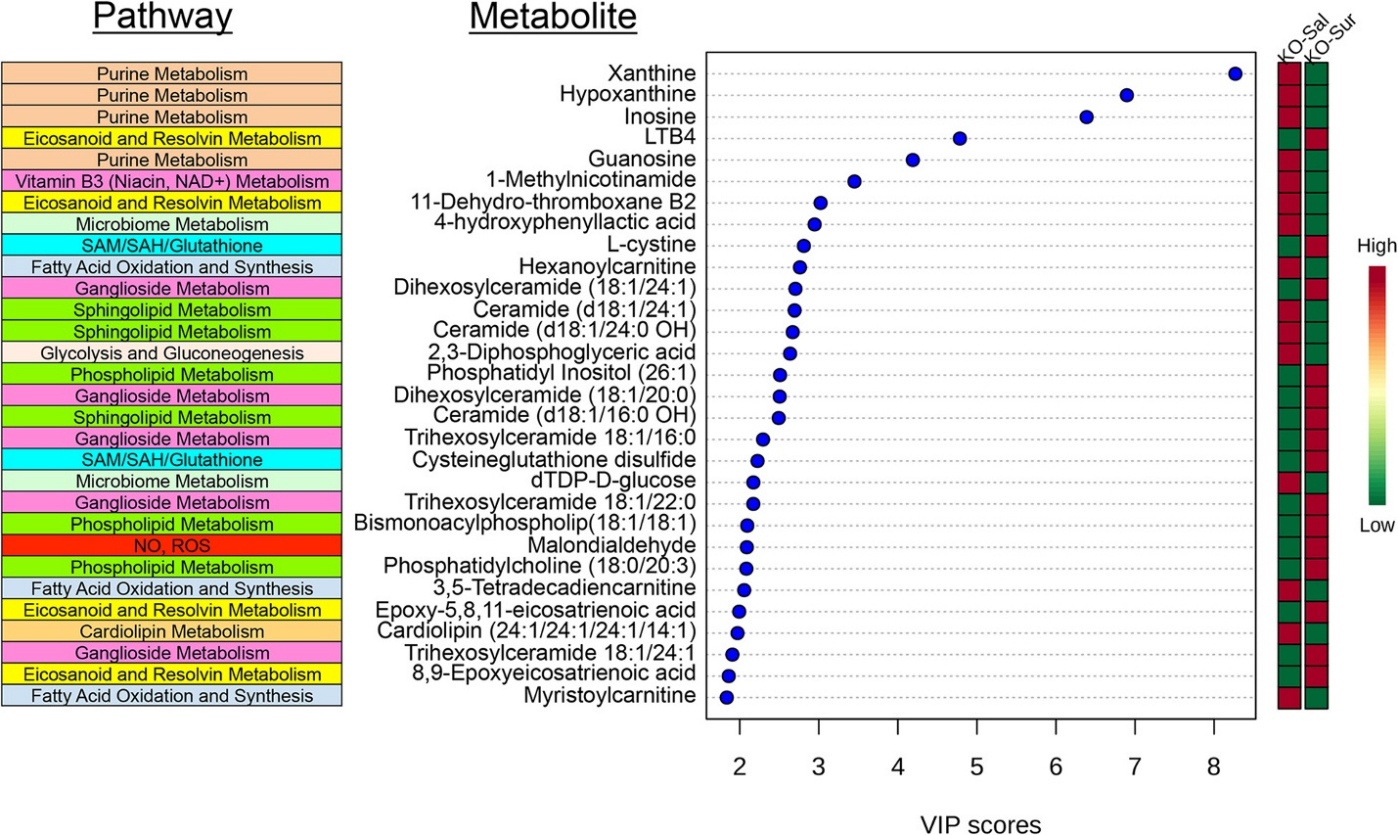
**Metabolites and pathways associated with suramin treatment in the Fragile X model.** The top 30 most discriminating metabolites and their biochemical pathways ranked by variable importance in projection (VIP) scores. See Additional file 1: Table S3 for a complete list of the top 58 discriminating metabolites. VIP scores ≥1.5 were considered statistically significant. Age 25 weeks, N = 9-11 per group.

We analyzed the metabolomic effects in plasma of Fragile X mice after weekly treatment with suramin or saline. We measured 673 metabolites from 60 pathways by mass spectrometry (Additional file 1: Table S2), analyzed the data by partial least squares discriminant analysis (PLSDA), and visualized the results by projection in three dimensions (Figure 5), and ranked by the metabolic changes by variable importance in projection (VIP) scores (Figure 6). This analysis focused on the rank order of importance. Larger sample sizes, usually ≥15 animals per group, are required for more comprehensive metabolomic statistical analysis [53]. We found that suramin produced pharmacometabolomic changes in one-third of the biochemical pathways interrogated (20 of 60 pathways). These are summarized below.

### Metabolic pathway analysis

The top 11 of 20 discriminating metabolic pathways were represented by two or more metabolites and contributed 89% of the most discriminating metabolites in the Fragile X mouse model treated with suramin (Table 1). These pathways were: purines (20%), fatty acid oxidation (12%), eicosanoids (11%), gangliosides (10%), phospholipids (9%), sphingolipids (8%), microbiome (5%), SAM/SAH glutathione (5%), NAD^+^ metabolism (4%), glycolysis (3%), and cholesterol metabolism (2%) (Table 1).

**Table 1:** **Biochemical pathways with metabolites changed by antipurinergic therapy in the Fragile X mouse model**

No.	Pathway name	Measured metabolites in the pathway (N)	Expected pathway proportion (P = N/673)	Expected hits in sample of 58 (P * 58)	Observed hits in the top 58 metabolites	Fold enrichment (Obs/Exp)	Impact (Sum VIP score)	Fraction of impact (VIP) explained (% of 136.0)	Suramin treatment effect (KO-Sur/KO-Sal)
1	Purine metabolism	41	0.061	3.54	5	1.41	27.2	20.0%	4/5 Decreased
2	Fatty acid oxidation and synthesis	39	0.057	3.37	9	2.67	16.8	12.4%	9/9 Decreased
3	Eicosanoid and resolvin metabolism	36	0.053	3.11	6	1.93	14.7	10.8%	4/6 Increased
4	Ganglioside metabolism	12	0.018	1.04	6	5.79	13.4	9.8%	6/6 Increased
5	Phospholipid metabolism	115	0.18	9.93	6	0.60	11.5	8.5%	6/6 Increased
6	Sphingolipid metabolism	72	0.105	6.21	5	0.80	11.1	8.2%	3/5 Decreased
7	Microbiome metabolism	33	0.047	2.85	3	1.05	6.7	4.9%	2/3 Decreased
8	SAM, SAH, methionine, cysteine, glutathione metabolism	22	0.032	1.90	3	1.58	6.7	4.9%	3/3 Increased
9	Vitamin B3 (Niacin, NAD+) metabolism	8	0.012	0.69	2	2.90	5.2	3.8%	1/2 Increased
10	Glycolysis and gluconeogenesis	18	0.026	1.55	2	1.29	4.2	3.1%	2/2 Decreased
11	Cholesterol, cortisol, non-gonadal steroid metabolism	29	0.042	2.50	2	0.80	3.2	2.4%	2/2 Increased
12	Nitric oxide, superoxide, peroxide metabolism	6	0.009	0.52	1	1.93	2.1	1.5%	Increased
13	Cardiolipin metabolism	12	0.018	1.04	1	0.97	2.0	1.4%	Decreased
14	Bile salt metabolism	8	0.012	0.69	1	1.45	1.8	1.3%	Increased
15	Branch chain amino acid metabolism	13	0.019	1.12	1	0.89	1.7	1.2%	Increased
16	Isoleucine, valine, threonine, or methionine metabolism	4	0.006	0.35	1	2.90	1.7	1.2%	Increased
17	Pyrimidine metabolism	31	0.051	2.68	1	0.37	1.6	1.1%	Decreased
18	Krebs cycle	17	0.025	1.47	1	0.68	1.6	1.1%	Increased
19	Vitamin B6 (pyridoxine) metabolism	5	0.007	0.43	1	2.32	1.5	1.1%	Increased
20	Pentose phosphate, gluconate metabolism	11	0.016	0.95	1	1.05	1.5	1.1%	Increased
	20 of 60 pathways dysregulated	532 (0.79 x 673)	79% (532/673)	46 (0.79 x 58)	58		136.0	100%	33/58 Increased

Pathways were ranked by their impact measured by summed VIP (Σ_VIP_; variable importance in projection) scores. A total of 58 metabolites were found to discriminate suramin-treated and saline-treated Fragile X knockout groups by multivariate partial least squares discriminant analysis (PLSDA). Significant metabolites had VIP scores of ≥1.5. Twenty (33%) of the 60 pathways interrogated had at least one metabolite with VIP scores ≥1.5. The total impact of these 58 metabolites corresponded to a summed VIP score of 136. The fractional impact of each pathway is quantified as the percent of the summed VIP score and displayed in the final column on the right in the table. Antipurinergic therapy with suramin not only corrected purine metabolism, but also produced changes in 19 other pathways associated with multi-system improvements in ASD-like symptoms.

**Figure 7:**
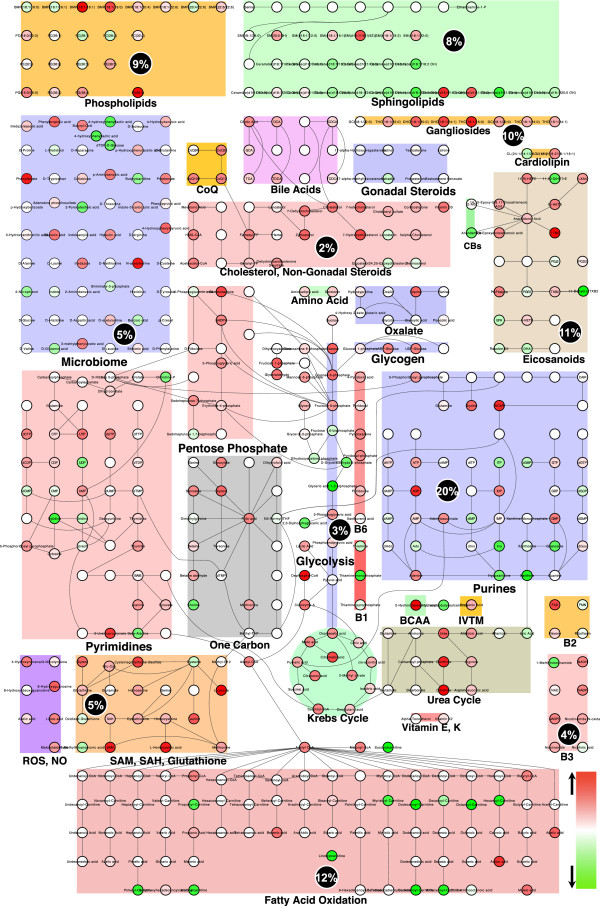
**Cytoscape visualization of the metabolic pathways altered by antipurinergic therapy in the Fragile X mouse model.** Twenty-six of the 60 biochemical pathways interrogated in our metabolomic analysis are illustrated. See Additional file 1: Tables S2 and S3 for complete listing of pathways and discriminating metabolites, respectively. The fractional contribution of each of the top 20 pathways altered by suramin treatment is indicated as a percentage of the total variable importance in projection (VIP) score in the black circles. Purine metabolism contributed 20% of the top ranked VIP scores, followed by fatty acid oxidation (12%), eicosanoids (11%), gangliosides (10%), phospholipids (9%), and 15 other biochemical pathways as indicated. Smaller circles indicate the metabolites in each pathway, quantified by z-score. Metabolites in red were increased. Metabolites in green were decreased by treatment with suramin. Age 25 weeks, N = 9-11 per group.

A simplified map of metabolism is illustrated in the form of 26 major biochemical pathways in Figure 7. This figure shows the effect of suramin treatment on each metabolite as measured in the plasma. The magnitude of the pharmacometabolomic effect is quantified as the z-score for nearly 500 metabolites. Inspection of this figure leads to several conclusions. First, 1-carbon folate and Krebs cycle metabolism are dominated by red shading, indicating a general increase in methylation pathways, and mitochondrial oxidative phosphorylation. Next, there was a generalized increase in intermediates of the SAM/SAH and glutathione metabolism. Purine metabolism showed a mixture of upregulated precursors of adenine nucleotides and downregulated inosine and guanosine precursors. There was a generalized increase in gangliosides, phospholipids, and cholesterol metabolites needed for myelin and cell membrane synthesis. Finally, there was a generalized decrease in nine of nine acyl-carnitine species. Acyl-carnitines accumulate when fatty acid oxidation is impaired, and decline when normal mitochondrial fatty acid oxidation is restored. Each of these pathways is a known feature of the cell danger response (CDR) [54].

### Lipid metabolism

Disturbances in lipid metabolism were a prominent feature of the Fragile X mouse model (Additional file 1: Figures S3A-D), and its response to treatment (Table 1, Figure 7). Treatment with suramin produced concerted effects in eight different classes of lipids that collectively explained 54% of the top ranked metabolites identified by multivariate analysis. In rank order of importance these were: fatty acid metabolism (12%), eicosanoid metabolism (11%), ganglioside metabolism (10%), phospholipid metabolism (9%), sphingolipids (8%), cholesterol/sterols (2%), cardiolipin (1%), and bile acids (1%) (Table 1). Suramin also had a significant impact on lipid metabolism in the MIA model. Four of the top six metabolic pathways were lipids, explaining 30% of the top ranked VIP scores. In rank order of importance the lipid pathways in the MIA model were: phospholipids (8%), bile acids (8%), sphingolipids (7%), and cholesterol/sterols (7%) [9].

### Shared metabolic pathways in the MIA and Fragile X models

**Figure 8:**
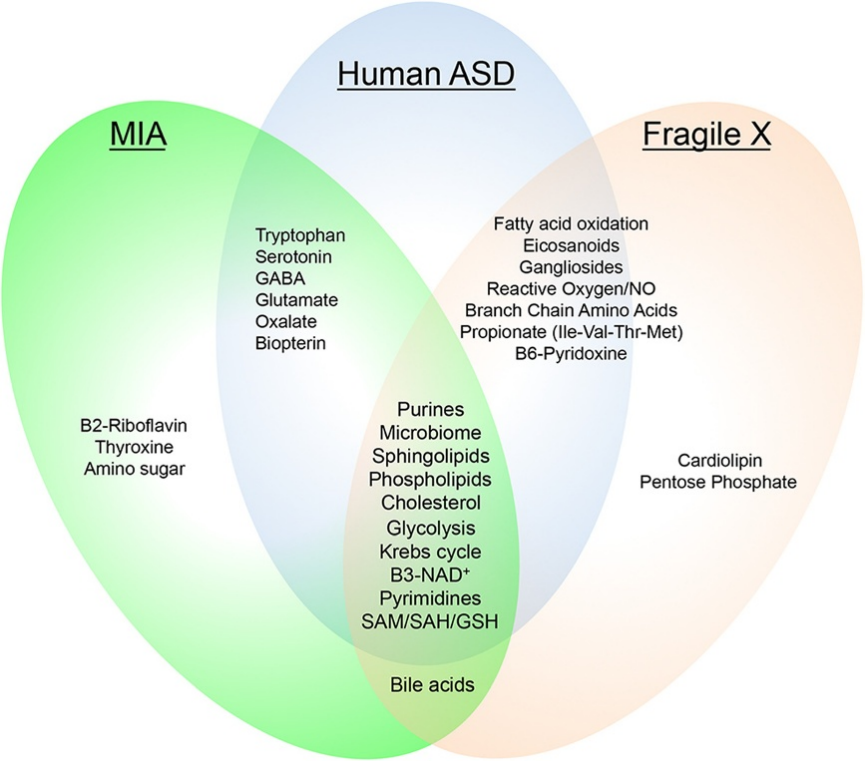
**Metabolic pathways shared by the MIA and Fragile X mouse models and human ASD.** Comprehensive metabolomic analysis identified 20 biochemical pathways associated with symptom improvements in the Fragile X (*Fmr1 knockout*) model. Seventeen pathways were shared with human ASD, and 11 were shared with the maternal immune activation (MIA) model of ASD. The 17 metabolic pathways shared with human ASD were: purines, microbiome, sphingolipids, phophoslipids, cholesterol/sterols, glycolysis, Krebs cycle, vitamin B3-NAD^+^, pyrimidines, S-adenosyl methionine (SAM)/S-adenosylhomocysteine (SAH)/glutathione (GSH), fatty acid oxidation, eicosanoids, gangliosides, reactive oxygen species and nitric oxide (ROS/NO), branched chain amino acids, propionate metabolism and propiogenic amino acids (Ile, Val, Thr, and Met), and vitamin B6-pyridoxine metabolism. Bile acids were common to both the Fragile X and MIA models, but have not yet been studied in human autism.

We compared the 20 pathways found to be altered in the Fragile X model (Table 1) to the 18 metabolic pathways that were altered in the maternal immune activation (MIA) model [8]. A Venn diagram of this comparison revealed 11 pathways that were shared between these two models (Figure 8). These were purines, the microbiome, phospholipid, sphingolipid, cholesterol, bile acids, glycolysis, the Krebs cycle, NAD^+^, pyrimidines, and S-adenosylmethionine (SAM), S-adenosyl-homocysteine (SAH), and glutathione (GSH) metabolism.

## Discussion

The Fragile X mouse model is one of the most commonly studied genetic mouse models of ASDs. Using this genetic model, we found that antipurinergic therapy (APT) with suramin improved the behavioral, metabolic, and the synaptic structural abnormalities. We previously showed that the ASD-like symptoms of the maternal immune activation (MIA) mouse model were also improved by antipurinergic therapy [8]. Regardless of the model - whether ‘environmental’ like the MIA model, or ‘genetic’ like the Fragile X knockout model - antipurinergic therapy with suramin corrected the abnormalities that characterized each model. Our results support the novel conclusion that antipurinergic therapy is operating by a mechanism that lies close to the root cause of the core behaviors and development in both the environmental MIA, and the genetic Fragile X models of ASD. This mechanism appears to be traceable to mitochondria and regulated by purinergic signaling.

We considered several caveats before drawing these conclusions. The Fragile X knockout mouse model is an imperfect model of human Fragile X syndrome. Human Fragile X syndrome is not the result of knockout of the gene, but rather an expansion of a CGG triplet repeat in the 5’ untranslated region of the gene, and variable phenotypes have been reported in the mouse model [26]. As with many syndromic, single gene disorders, human Fragile X syndrome itself is an imperfect model of non-syndromic ASDs. At least 40% of the boys [55], and over 90% of the girls [56] with Fragile X syndrome do not meet the diagnostic criteria for ASD. We studied the most commonly reported genotype of the *Fmr1* knockout mouse model, which was backcrossed for 11 generations on the genetic background of the FVB strain of laboratory mouse. We compared the behavioral features of this *Fmr1* knockout to the FVB control strain. A potential weakness of our study is that *Fmr1* knockout and FVB control strains are not littermate controls raised by the same mothers. Some behavioral differences might be the result of differences in maternal genotype and rearing. However, the point of our study was not to re-confirm the known behavioral and molecular features of Fragile X model, but rather to ask the question, ‘Are the abnormalities treatable with antipurinergic therapy (APT)?’ Our data suggest that they were.

Other treatments have also been successful in mitigating the symptoms of the Fragile X mouse by addressing specific neurotransmitter or synaptic defects. These have included drug inhibition of glutamatergic signaling with mGluR5 inhibitors [57], inhibition of endocannabinoid signaling [41], and genetic inhibition of amyloid β precursor protein (APP) [58]. A number of metabolic therapies have also been successful. These have included acetyl-L-carnitine [59], omega 3 fatty acid therapy [60], and inhibition of the metabolic control enzyme glycogen synthase kinase 3β (GSK3β) [32]. Remarkably, we found that antipurinergic therapy addressed each of these abnormalities with a single intervention. Minocycline has also shown benefit in both human Fragile X syndrome [61] and mouse models [62]. Interestingly, many of the neuroprotective and anti-inflammatory effects of minocycline have been traced to its actions on mitochondrial function [63, 64], and may also act to decrease hyperpurinergia by moderating mitochondrial ATP synthesis.

Purine metabolism was the most discriminating single metabolic pathway in the Fragile X mouse model treated with suramin, contributing 20% of the top ranked metabolites identified by multivariate analysis (Table 1, Figure 7). An important pharmacologic mechanism of action of suramin is as a competitive antagonist of extracellular ATP and other nucleotides, acting at purinergic receptors [9, 65]. Suramin also has nearly 30 other actions [18]. Our metabolomic data show that the major functional impact of suramin in the Fragile X mouse model was on purine metabolism (Table 1). Purinergic signaling abnormalities linked to autism-like behaviors are not restricted to animal models. Several inborn errors in purine [15] and pyrimidine metabolism [14] are well known to be associated with autism-like behaviors [9]. In addition, abnormalities in purine metabolism leading to hyperuricosuria in 20% of children with non-syndromic autism have been described [66]. The specific link between purine metabolism in ASD and purinergic signaling was first made in 2012 [67], and tested in the MIA mouse model in 2013 [8]. Interestingly, brain purinergic signaling was recently identified as one of the top gene expression pathways correlated with abnormal behaviors in children with ASD [68].

We next compared the metabolomic results for both the maternal immune activation (MIA) [9] and Fragile X mouse models of ASD (Figure 8). We found 11 metabolic pathways that were common to both models. These were purines, microbiome, phospholipids, sphingolipids/gangliosides, cholesterol/sterol, bile acids, glycolysis, mitochondrial Krebs cycle, NAD^+^/H, pyrimidines, and S-adenosylmethionine/homocysteine/glutathione (SAM/SAH/GSH) metabolism. Seventeen of the 20 metabolic pathway disturbances found in the Fragile X mouse model have been described in human ASD. These include purine metabolism [15, 66], fatty acid oxidation [69], microbiome [70, 71], phospholipid [72], eicosanoid [73–75], cholesterol/sterol [76], sphingolipids and gangliosides [77, 78], glycolysis, Krebs cycle and mitochondrial metabolism [79–81], nitric oxide and reactive oxygen metabolism [82], branched chain amino acids [83], propionate and propiogenic amino acid metabolism (IVTM; Ile, Val, Thr, Met) [84], pyrimidines [14], SAM/SAH/glutathione [85], vitamin B3-NAD^+^ metabolism [86], and vitamin B6-pyridoxine metabolism [87]. We found plasma cardiolipin was therapeutically downregulated by suramin. Although elevations in plasma cardiolipin species have not yet been reported in children with autism, anti-cardiolipin antibodies have [88].

The upregulation of glycolysis and downregulation of mitochondrial Krebs cycle seen in the Fragile X model of ASD are a direct consequence of the regulated decrease in mitochondrial oxidative phosphorylation (oxphos). This was corrected by suramin (Figure 7). The conditions of increased substrate supply and decreased utilization, create a poised state of mitochondrial underfunction associated with increased reserve capacity. When basal mitochondrial oxygen consumption is decreased, cellular heat production from mitochondria is reduced. This can lead to a decrease in basal body temperature. We observed a 0.7°C decrease in basal body temperature in the Fragile X mice (Figure 1G). A similar decrease was seen in the MIA mouse model [8]. A poised increase in mitochondrial reserve capacity can also produce primed mitochondria with the capacity to respond explosively to stress. In some cases, this is manifest as a large increase in mitochondrial reactive oxygen species (ROS) production. Interestingly, an increase in mitochondrial reserve capacity and increased ROS production under stress has been documented in 32% of lymphocytoblastoid cell lines (8/25 = 32%; 95% CI 15-54%) derived from children with ASD [79]. When an explosive discharge of mitochondrial ROS occurs transiently, uncoupling can result, leading to a large increase in mitochondrial heat production and high fevers. Superfevers of 104.5° to 105.5°F have been described in occasional patients with Fragile X during infectious illness (personal communication, Randi J. Hagerman).

When cellular resources are redirected away from work, changes in activity-dependent gene expression result in a reduction in unused proteins [89]. With time these underutilized cells lose the capacity for specific kinds of work and can enter a physiologically-induced hypometabolic state that protects the cell from harsh extracellular conditions and promotes cellular persistence. This state shares metabolic similarities to the *dauer* state in *C. elegans*[90], embryonic diapause in mammals [91], plant seed development [92], and stem cells that can be recruited back into cycle after stasis or injury [93]. This latter point suggests that tissues and organs can exist as shifting mosaics of fully active cells, and hypometabolic cells that can be called into action, depending on environmental conditions. In each case, mitochondrial fatty acid oxidation is decreased to facilitate intracellular lipid accumulation needed for persistence metabolism. Fatty acid oxidation was the second most discriminating pathway in the Fragile X mouse model treated with suramin, contributing 12% of the top ranked metabolites identified by multivariate analysis (Table 1, Figure 7). Several acyl-carnitine species were elevated (Table 1, Figure 6). This is a hallmark of diminished mitochondrial fatty acid oxidation [94]. Similar elevations of acyl-carnitines have been reported in 17% of human ASD [69]. Suramin treatment decreased plasma acyl-carnitine levels in the Fragile X model (Figures 6 and 7).

Eicosanoid metabolism was the third most discriminating metabolic pathway in the Fragile X mouse model treated with suramin, contributing 11% of the top ranked metabolites identified by multivariate analysis (Table 1, Figure 7). Eicosanoid metabolism plays a crucial role in regulating the balance of inflammation and anti-inflammation after acute injury, during chronic disease [95, 96], and in the antiviral and antibacterial innate immune response [97]. Upregulated lipoxygenase activity in the Fragile X mouse model was recently predicted on the basis of FMRP binding to lipoxygenase mRNA, and disinhibition in the knockout, in a medical hypothesis paper by Beaulieu [75]. The predicted increase in hydroxyeicosatetraenoic acid (HETE) species would support mGluR5-mediated long-term depression (LTD) [98], which is a well-known problem in Fragile X syndrome [99]. We found that suramin treatment increased two epoxyeicosatrienoic acids (EETs), and decreased a stable metabolite of platelet thromboxane A2 (TXA2), 11-dehydro-thromboxane B2 (Figures 6 and 7). The increase in EETs has several physiologic benefits. These include a decrease in ER stress [100], an increase in AMPK activation, and an increase in autophagy [101]. The increase in EETs and decrease in TXA2 were consistent with the anti-inflammatory action of antipurinergic therapy with suramin.

Considered as a coordinated system, these data show that the metabolic disturbances in the mouse models of ASD are similar to those found in human ASD (Figure 8). The data provide strong support for the biochemical validity of both the MIA and Fragile X mouse models. In addition, the metabolomics data revealed for the first time the surprising observation that the environmental MIA and the genetic Fragile X mouse models, and human ASD, all shared disturbances in biochemical pathways previously identified as features of the evolutionarily conserved cell danger response (CDR) (Figure 8) [54]. We found that in the mouse models, both the ASD-like symptoms and the biochemical features of the CDR were corrected by antipurinergic therapy with suramin.

Purinergic signaling begins with the regulated release of purine nucleotides like ATP, or pyrimidines like UTP, through channels in the cell membrane for autocrine and paracrine signaling, and by vesicular fusion during neurotransmission [102, 103]. Purinergic (P2X and P2Y) receptors bind to extracellular ATP and other nucleotides as a means of sensing cellular health and danger [54, 104]. In this usage, the word ‘danger’ is not an anthropomorphic construct. Danger has a chemical meaning in cells that equates to metabolic mismatches between substrate/product ratios and the gene-coded and allosterically regulated equilibrium constant (K_eq_) of each relevant enzyme located in mitochondria, and the conductances of transporters in and out of the organelles [105]. These mismatches are coupled to mitochondrial oxygen consumption, electron flow, redox, and oxidative phosphorylation, and produce a sequence of graded metabolic responses that alter DNA methylation [106], histone modifications [107], and lead to new cellular states of gene expression and function [108]. In most differentiated cells, mitochondria make 90% of the ATP, and process 90% of the carbon skeletons and activated sulfur intermediates used to create the building blocks for cell growth, function, detoxification, and repair. Mitochondria serve as the cellular translators of real-time metabolic information, integrating it with the genetics of the cell, and providing feedback in the form of retrograde signals to the nucleus [109] used to change gene expression.

When extracellular ATP binds to purinergic receptors on the cell surface it is participating in what we call a ‘long-path’ retrograde signaling circuit from mitochondria to the unstirred water layer on the cell surface, to neighboring cells, or back through autologous cell membrane G-protein coupled receptors and ion channels, to calcium signaling, back to mitochondria and other cellular compartments, and ultimately to the nucleus, changing gene expression. This extracellular purinergic signaling circuit is well known to regulate innate immunity, oxidative stress and shielding [110], inflammation, and cytokine production [104], sensory perception [111], in addition to sleep, cognition [112], and the autonomic nervous system [113]. Intracellular adenine nucleotides like NAD^+^/H, NADP^+^/H, cyclic-ADP-ribose (cADPR), and nicotinic acid adenine dinucleotide phosphate (NAADP) are also traceable to mitochondria, are interconverted by the enzyme CD38 [114], and play important roles in a ‘short-path’ retrograde signaling circuit that regulates redox, calcium release, sodium and potassium channels [115], synaptic long-term depression [116], autophagy [117], defense against intracellular pathogens [118], and social behavior by modulating oxytocin secretion [119]. These studies underscore the surprising role of extracellular and intracellular purines in regulating a diverse array of biological phenomena, ranging from innate immunity and cellular defense, to sleep, cognition, behavior, perception, affect, memory, and learning.

Although our results showing the correction of ASD-like behaviors, and improvements in metabolism and brain synaptic abnormalities in the Fragile X mouse model are encouraging, there are several caveats that must be considered before extending the results to humans. First, while the Fragile X mouse model captures several features of ASD, no animal model can fully capture the complexities of human behavior. Second, suramin is a poor drug choice for chronic use because of potentially toxic side effects that can occur with prolonged treatment [120]. Third, human forms of ASD may occur by mechanisms not captured by the Fragile X model. Mechanisms that do not involve the pathological persistence of the cell danger response (CDR) [54] may not be amenable to antipurinergic therapy. Newer, safer, more selective antipurinergic drugs, and human clinical trials will be necessary to answer these questions.

## Conclusions

The data reported in this study show that the efficacy of antipurinergic therapy cuts across disease models of ASD. Both the environmental MIA [8, 9] and the genetic Fragile X models (Figures 1, 2, 3, 4, 5, 6, 7, 8, Tables 1 and 2) responded with complete, or near-complete, resolution of symptoms, even when treatment was not begun until adolescence, or adulthood. The data support the hypothesis that disturbances in purinergic signaling may be a common denominator and effective therapeutic target in both the environmental MIA and genetic Fragile X mouse models of autism spectrum disorders.

**Table 2:** **Summary of antipurinergic therapy results in the Fragile X mouse model of autism spectrum disorders**

Feature	Abnormality in Fragile X males vs. FVB controls	Response to antipurinergic therapy
Social preference	Decreased	Normalized (*P* <0.05)
Social novelty	Decreased	Normalized (*P* <0.05)
T-maze spontaneous alternation	Decreased	Normalized (*P* <0.001)
Marble burying	Decreased	Normalized (*P* <0.05)
Core body temperature	Decreased	Normalized (*P* <0.001)
Acoustic startle	Decreased [sic]	Unchanged (*P* = ns)
Metabolomics (60 pathways measured)	20 of 60 pathways disturbed	33 metabolites increased and 25 decreased of 58 changed
Synaptosomal structure by electron microscopy	Fragile and malformed post-synaptic densities; Accumulation of electron dense matrix material	Improved
Synaptosomal proteins (54 interrogated)	N/A	17 proteins were changed (for example, decreased PI3/GSK3β, GluR1, C1q, and APP; increased PPARβ/δ)
Locomotor activity - total distance (hyperactivity), center exploration, holepokes, rearing	None	N/A

## Electronic supplementary material

Additional file 1: **This supplement includes a single PDF file with: supplementary Results, Methods, References, three tables, and four figures.** **Figure S1.** Confirmation of Fragile X protein expression knockout in the *Fmr1*/FVB Mouse Model. **Figure S2.** Acoustic startle and prepulse inhibition. **Figure S3.** Acyl-carnitine studies in *Fmr1* knockout mouse models. **Figure S4.** Western blot assay linearity and precision analysis. **Table S1.** Synaptic proteins interrogated and antibodies used. **Table S2.** Biochemical pathways and metabolites interrogated. **Table S3.** Metabolites changed by antipurinergic therapy in the Fragile X model. (PDF 811 KB)

## Abbreviations

APP: Amyloid β precursor protein
APT: Antipurinergic therapy
ASD: Autism spectrum disorders
C1q: Complement factor 1 subunit q
CDR: Cell danger response
*Fmr1*: Fragile X mental retardation gene locus 1
IP3: Inositol triphosphate
MIA: Maternal immune activation
P2X: Ionotropic purinergic receptors
P2Y: G-protein coupled purinergic receptors
TDP43: Tar DNA binding protein 43.
