# Supplementary material for: Antipurinergic therapy corrects the autism-like features in the Fragile X (Fmr1 knockout) mouse model
Source: Mol Autism. 2015 Jan 13;6:1. doi: 10.1186/2040-2392-6-1 (PMC4334917; doi:10.1186/2040-2392-6-1)
Supplement: Supplementary file 1 — Additional file 1: This supplement includes a single PDF file with: supplementary Results, Methods, References, three tables, and four figures. Figure S1. Confirmation of Fragile X protein expression knockout in the Fmr1/FVB Mouse Model. Figure S2. Acoustic startle and prepulse inhibition. Figure S3. Acyl-carnitine studies in Fmr1 knockout mouse models. Figure S4. Western blot assay linearity and precision analysis. Table S1. Synaptic proteins interrogated and antibodies used. Table S2. Biochemical pathways and metabolites interrogated. Table S3. Metabolites changed by antipurinergic therapy in the Fragile X model. (PDF 811 KB) [file 13229_2014_156_MOESM1_ESM.pdf]

Additional File 1

Additional File 1. This supplement includes a single PDF file with: supplementary Results, Methods, References, three tables, and four figures.

Supplementary Results

Incidental Note of Decreased Acyl-Carnitines in FVB Wild-type Controls

In the course of our studies comparing the Fragile X knockout animals to wild-type FVB controls, we found an apparent fatty acid oxidation defect that was characterized by a 2-7 fold increase in acyl-carnitine esters in the plasma (Additional File 1: Figure S3A-D). However, when we evaluated the same *Fmr1* knockout allele on a C57BL6/J (B6) background, we discovered that the actual difference was not due to the knockout per se. Instead, we found an unusually low level of acyl-carnitines in the FVB controls compared to B6 wild-type controls (Additional File 1: Figure S3B). This created the appearance of elevated carnitines in the *Fmr1* knockout on the FVB background, even though the quantitative levels were nearly identical in all animals with Fragile X, regardless of the genetic background (Additional File 1: Figure S3A-D).

Decreased Acyl-Carnitine Levels in the FVB Wild-type Controls

Metabolomic analysis revealed a 2-7-fold increase in plasma acyl-carnitine esters in the FMR knockout when compared to the FVB background controls (Additional File 1: Figure S3A). This pattern appeared at first to be a forme fruste of Multiple Acyl-CoA Dehydrogenase Deficiency (MADD), also known as glutaric aciduria type II (GAII). MADD is an inborn error of fatty acid oxidation. It results from mutations in the proteins that transfer electrons from mitochondrial fatty acid, branched chain amino acids, and lysine and tryptophan oxidation to the mitochondrial electron transport chain (ETFQO), or from mutations in a carrier protein for the cofactor riboflavin [1]. Severe forms of MADD produce a characteristic 5-500-fold increase in plasma

acyl-carnitine esters spanning four to 20 carbons (C4-C20) in length, resulting in many acyl-carnitine concentrations greater than 100 µM. The clinical features of MADD are variable, ranging from overwhelming acidosis and death in the first few days of life [2], to riboflavin-responsive muscle weakness presenting after 25 years of age [3]. Non-monogenic, seasonal and dietary forms of MADD are known in horses [4], and can be caused by riboflavin deficiency in rodents [5]. The biochemical phenotype in the FVB/*Fmr1* knockout mice was an imperfect match to MADD, not only because it was quantitatively milder, but also because it was qualitatively different. For example, glutarylcarnitine is not elevated in the *Fmr1* knockout (Additional File 1: Figure S2a), but is elevated in authentic Glutaric Aciduria Type II (GAII; MADD). In addition, unesterified carnitine (C0), acetyl (C2), and propionyl (C3) carnitines were elevated in the FMR knockout mouse (Table 2), but are more typically decreased or normal in authentic MADD [6].

To investigate the MADD-like phenotype, we first looked for evidence of a defect in riboflavin metabolism as reflected by differences in riboflavin, FMN and FAD in the plasma. Riboflavin and FMN were normal in the *Fmr1-KO*/FVB mice (data not shown). FAD was elevated by 53% in the *Fmr1-KO*/FVB animals compared to FVB controls, and was further increased by suramin treatment. These data suggested that the increase in plasma FAD was a beneficial response to a relative deficiency in mitochondrial fatty acid oxidation caused by the *Fmr1* knockout on the FVB background, and not the cause of the MADD-like acyl-carnitine profile. We next confirmed that the *Fmr1* knockout did not produce a secondary defect in ETFQO expression by western analysis (data not shown). We next purchased *Fmr1* knockout animals bred onto the C57BL/6J background and repeated the metabolomic studies on the *Fmr1-KO*/B6 animals compared to B6 controls. The results were surprising. We found that FVB wild-type control animals have acyl-carnitine levels that are 2-60 fold lower than C57BL/6J wild-type control animals (Figure S3B). The absolute concentrations of the plasma acyl-carnitines in both *Fmr1* knockouts (FMR/FVB

and FMR/B6) were virtually indistinguishable (Figure S3C). However, because the FVB control strain has acyl-carnitine concentrations that are 2-60 fold lower than B6 controls, the *Fmr1* knockout produced an apparent forme fruste of the MADD phenotype when compared to the FVB control strain. When the *Fmr1* knockout was examined on the C57BL/6J background, there was no MADD-like phenotype (Additional File 1: Figure S3D). These data show that the same mutation (*Fmr1* knockout) produces a different metabolic phenotype on different genetic backgrounds.

Acoustic Startle Was Decreased in Knockout and Unchanged by Suramin

Fragile X mice fail to show the normal developmental increase in acoustic startle with age. When measured after 3 months of age, they are less sensitive to acoustic startle than controls despite normal hearing [7]. We found that the Fragile X mice had startle magnitudes at pulse intensities of about 100-110db that were 44% lower than wild-type. Suramin did not change the startle magnitude in *Fmr1* knockout or FVB control mice (Additional File 1: Figure S2A).

We also measured prepulse inhibition of startle (PPI) in the startle session. PPI exploits the observation that a soft sound (prepulse) delivered 50 msec or more before a loud sound will reduce the startle magnitude measured as jump force compared to the loud sound given alone. This is widely studied as a measure of sensorimotor gating, but literature reports of PPI abnormalities in the Fragile X mouse model have been mixed and age-dependent [7-10]. No consistent PPI differences were observed between *Fmr1* knockouts and controls. Suramin had no effect on PPI (Additional File 1: Figure S2B).

Locomotor Activity

Locomotor activity, hyperactivity measured as total distance traveled, hole poke exploration, and vertical investigative behavior (rearing) were quantified by automated beam break analysis in

the mouse behavioral pattern monitor (mBPM) [11]. No significant differences were found between the Fragile X knockout model and controls, or between saline treatment and suramin (data not shown).

Supplementary Methods

Social Preference and Novelty

Social preference and novelty were tested using a three-chambered box as previously described [12], with modifications designed to examine novel stranger interactions. Briefly, a Plexiglas box (60cm L x 60cm W x 30cm H) was divided into 3 equal compartments by Plexiglas partitions containing an opening through which the mice could freely enter the 3 chambers. All testing was performed between the hours of 8 am and 1 pm. The test was conducted in three 5-minute phases. In the habituation phase (phase I), the test mouse was allowed to explore the empty chambers for 5 minutes. In the social preference phase (phase II), a stainless steel wire cup (Galaxy Cup, Spectrum Diversified Designs, Inc., Streetsboro, OH) was placed into each of the two outer chambers. The test mouse was briefly removed and an unfamiliar mouse, age and sex matched, was placed under one of the wire cups. The test mouse was then gently placed back in the arena and given an additional 5 minutes to explore. In the social novelty phase (phase III), each mouse was further tested in a third 5-minute session to quantitate preference to spend time with a new stranger. The test mouse was briefly removed, and a new unfamiliar mouse was placed under the wire cage that had been previously empty. The test mouse thus chooses between the first already-investigated and now familiar mouse, and the novel unfamiliar mouse (stranger 2). Room lighting for social behavior studies was 1-2 lux, measured using a Minolta IV F light meter. An overhead camera (Sony CCD Digital Ultra Pro Series, able to detect images down to 0.05 lux) and Ethovision v3 video tracking software (Noldus, Leesburg VA) were used to record the amount of time spent in each chamber and the number of entries

into each chamber. In addition, a human observer, blinded to the treatment groups, scored time spent sniffing each wire cage, using Ethovision Observer software. Only male mice were tested. Stranger mice were habituated to a wire cup for at least 30 minutes before use. Stranger mice were used up to 4 times before new strangers were cycled into the experiment. The location (left or right) of the stranger 1 and stranger 2 mouse alternated across subjects. Results of social behavior testing are reported as the percent of time spent interacting with a stranger mouse vs empty cup in phase II (social preference), and as the percent of time spent interacting with the familiar mouse (stranger 1) vs the unfamiliar mouse (stranger 2) during phase III (social novelty).

T-Maze

The T-maze apparatus is constructed of black plexiglass. The protocol is adapted from Frye and Walf [13]. The main stem is 45 cm long, 10 cm wide, and 24 cm high. Each side arm is 35 cm long, 10 cm wide, and 24 cm high. The side arms are separated from the stem by horizontal sliding doors. A start box, 8 cm in length, is also separated by a horizontal sliding door. Testing was conducted by an examiner that was blinded to the experimental groups, under low illumination, between 8 am and 1 pm. Only male animals were tested. Each mouse was tested in a session of 11 successive trials. The mice were not habituated to the maze. For the first trial only, one goal arm was closed off, forcing the mouse to choose the only open arm. Subsequent trials were by free choice. The chosen arm, and the time it takes for the mouse to choose (latency) were recorded. There was no confinement time in the chosen arm or in the start box. The percentage of alternated choices (mean +/- SEM) is reported.

Acoustic Startle and Prepulse Inhibition

Startle and PPI testing were performed in commercial startle chambers (SRLABsystem, San Diego Instruments, San Diego, CA). Within each chamber there was a Plexiglas cylinder (3.7cm

in diameter) into which the animal was placed. Sudden movements by the mouse were detected by a piezoelectric accelerometer attached below the cylinder. A loudspeaker provided the broadband background noise and acoustic stimuli, and the whole apparatus was housed within the ventilated, sound-attenuating chamber (39cm **x** 38cm **x** 58cm). A standard computer controlled stimulus presentations and response measures. The experimental session consisted of a 5 min acclimatization period to a 65 dB background noise (continuous throughout the session). During the session, 17 trial types were presented: six 40ms startle pulses (80, 90, 100, 110, or 120db; pulse alone); a no stimulus trial (nostim); five 20ms prepulse + pulse combinations [67,69, 73, or 81 dB prepulses followed 100ms later by a P120 stimulus, or 73 dB prepulse followed 100 ms later by a P105 stimulus; prepulses + pulse]; five 20ms prepulse + pulse combinations with varying inter-stimulus intervals [73 dB prepulse followed 20, 70, 120, 360, or 1080ms later by a P120 stimulus; prepulses (vISI) + pulse]. Trial types were presented in a varied order (5 presentations of each pulse alone trial, 5 presentations of each prepulses + pulse combination, 5 presentations of each prepulses (vISI) + pulse combination, and nostim trials occurring between each trial) with an average inter-trial interval (ITI) of 15 s. In addition, 5 of the pulse alone trials, which were not included in the calculation of PPI values, were presented at the beginning of the test session to achieve a relatively stable level of startle reactivity for the reminder of the session (based on the observation that the most rapid habituation of the startle reflex occurs within the first few presentations of the startling stimulus [66]. Another 4 of the pulse alone trials, which were also not included in the calculation of PPI values, were presented at the end of the test session to assess startle habituation.

Marble Burying

Marble burying was used to quantify spontaneous digging as a measure of a normal, genetically determined trait in rodents that has been shown to be uncorrelated with classical measures of anxiety [14]. Standard polycarbonate mouse cages (7.5” x 11.5” x 5”) were used without metal

fittings. Each cage was filled with 1/8-inch sieve corncob bedding to a depth of 2.5 inches. Twenty glass marbles (1 cm diameter) were placed in 4 evenly spaced rows of 5 on top of the bedding. The mice were habituated to the testing room for 30 minutes, and then each mouse was placed individually into a marble-containing cage. Testing was conducted in a semi-dark room. After 30 minutes, the number of buried and unburied marbles was counted. Marbles that were at least 2/3 covered with bedding were counted as buried.

Mouse Behavioral Pattern Monitoring

Ten mouse BPM chambers were used to assess spontaneous exploratory behavior as described previously [15]. Each chamber was illuminated from a single source of red light above the arena. The arena had dimensions of 30.5cm×61cm×38cm and was equipped with a Plexiglas holeboard floor with 3 floor holes and 8 wall holes. Holepoking behavior was detected using an infrared photobeam. The location of the mouse was recorded every 0.1 s using a grid of 12×24 infrared photobeams that were located 1 cm above the floor. The position of the mouse was assigned to 9 unequal regions described by a tic-tac-toe pattern. Rearing behavior was recorded using an array of 16 infrared photobeams 2.5cm above the floor aligned with the long axis of the chamber. At the start of each test session, mice were placed in the bottom left hand corner of the chamber, facing the corner and the test session started immediately. Four main factors were investigated: locomotor activity as measured by transitions (calculated as a movements between the 9 regions); surface investigatory behavior as measured by holepoking; vertical investigatory activity was measured as total rearing; and center entries were quantified.

Western Blot Assay Validation

We confirmed the linearity and quantitative precision of the Western blot assays as follows. First, we selected the most abundantly expressed protein that had the highest signal intensity by Western ECL analysis, and the least abundant protein that was altered by suramin treatment.

These proteins were pGSK3β(Ser9) (Figure 3) and StAR (Figures 3, 4P), respectively. We next confirmed that these proteins were being measured within the linear dynamic range of the assay by performing serial dilutions of synaptosomes loaded in each lane from 5 µg/lane to 20 µg/lane, comparing the ECL signal intensity curves. Least squares regression analysis showed that the assays were within the linear range for both proteins (Additional File 1: Figure S4AB). We also confirmed the linear dose response of P2Y1 and P2X3, two other proteins with signal intensities that were intermediate between GSK3β and StAR (Additional File 1: Figure S4CD). Second, we repeated the assays for these proteins on 3 separate days, using independently loaded SDS-PAGE gels and blots. The ratiometric precision (coefficient of variation = SD/mean) of the observed KO-suramin/KO-saline results was +/-15% (Additional File 1: Figure S4E). Two-way analysis of variance confirmed that between-day variation contributed only 0.3-1% of the total assay variance (Additional File 1: Figure S4F).

Notes on Non-Littermate Controls

Although genetically appropriate and widely used in treatment studies [16], the use of non-littermate, wild-type background mouse strains as controls for knockout animals has significant limitations for metabolomic and behavioral studies. The commercially available FVB control strain for the Fragile X mice has been bred separately for over 8 years since the original 11 backcrosses used to transfer the *Fmr1* knockout to the FVB strain background in 2006. In addition to genetic drift, the maternal metabolic environment is different in homozygous wild-type (X^+/X^+) dams compared to homozygous *Fmr1* knockouts (X^o/X^o). The different gestational metabolic environments can have both epigenetic and metabolic effects on the offspring that can interact with the direct effects of the knockout. The use of littermate controls produced by mating heterozygous (X^+/X^o) dams with wild-type (X^+/Y) sires overcomes this problem, but adds significantly to the duration and cost of the experiments, and does not answer questions

directed at the knockout response to treatment. We report the efficacy of suramin treatment to improve social behavior, metabolism, and synapse structure in the context of the Fragile X model. Future studies will be needed to directly compare suramin effects in wild-type littermate controls.

Supplementary References

1. Yonezawa A, Inui K: **Novel riboflavin transporter family RFVT/SLC52: identification, nomenclature, functional characterization and genetic diseases of RFVT/SLC52.** *Molecular aspects of medicine* 2013, **34:**693-701.
2. Nyhan WL, Barshop BA, Al-Aqeel AI: **Multiple acyl-coA dehydrogenase deficiency/glutaric aciduria type II/ethylmalonic-adipic aciduria.** In *Atlas of Inherited Metabolic Diseases, 3rd edition.* Edited by Nyhan WL, Barshop BA, Al-Aqeel AI. London, England: Hodder Arnold; 2012: 316-324
3. Xi J, Wen B, Lin J, Zhu W, Luo S, Zhao C, Li D, Lin P, Lu J, Yan C: **Clinical features and ETFDH mutation spectrum in a cohort of 90 Chinese patients with late-onset multiple acyl-CoA dehydrogenase deficiency.** *Journal of inherited metabolic disease* 2013.
4. van der Kolk JH, Wijnberg ID, Westermann CM, Dorland L, de Sain-van der Velden MG, Kranenburg LC, Duran M, Dijkstra JA, van der Lugt JJ, Wanders RJ, Gruys E: **Equine acquired multiple acyl-CoA dehydrogenase deficiency (MADD) in 14 horses associated with ingestion of Maple leaves (Acer pseudoplatanus) covered with European tar spot (Rhytisma acerinum).** *Molecular genetics and metabolism* 2010, **101:**289-291.
5. Goodman SI: **Organic aciduria in the riboflavin-deficient rat.** *The American journal of clinical nutrition* 1981, **34:**2434-2437.
6. Sahai I, Garganta CL, Bailey J, James P, Levy HL, Martin M, Neilan E, Phornphutkul C, Sweetser DA, Zytkovicz TH, Eaton RB: **Newborn Screening for Glutaric Aciduria-II: The New England Experience.** *JIMD reports* 2013.
7. Yun SW, Platholi J, Flaherty MS, Fu W, Kottmann AH, Toth M: **Fmrp is required for the establishment of the startle response during the critical period of auditory development.** *Brain research* 2006, **1110:**159-165.
8. Chen L, Toth M: **Fragile X mice develop sensory hyperreactivity to auditory stimuli.** *Neuroscience* 2001, **103:**1043-1050.
9. Frankland PW, Wang Y, Rosner B, Shimizu T, Balleine BW, Dykens EM, Ornitz EM, Silva AJ: **Sensorimotor gating abnormalities in young males with fragile X syndrome and Fmr1-knockout mice.** *Molecular psychiatry* 2004, **9:**417-425.
10. Renoux AJ, Sala-Hamrick KJ, Carducci NM, Frazer M, Halsey KE, Sutton MA, Dolan DF, Murphy GG, Todd PK: **Impaired sensorimotor gating in Fmr1 knock out and Fragile X premutation model mice.** *Behavioural brain research* 2014, **267:**42-45.
11. Halberstadt AL, van der Heijden I, Ruderman MA, Risbrough VB, Gingrich JA, Geyer MA, Powell SB: **5-HT(2A) and 5-HT(2C) receptors exert opposing effects on locomotor activity in mice.** *Neuropsychopharmacology : official publication of the American College of Neuropsychopharmacology* 2009, **34:**1958-1967.

12. Naviaux RK, Zolkipli-Cunningham Z, Nakayama T, Naviaux JC, Le T, Wang L, Schuchbauer M, Rogac M, Li Q, Dugan LL, Powell S: **Antipurinergic Therapy Corrects the Autism-Like Features in the Poly(IC) Mouse Model.** *PloS one* 2013.
13. Frye CA, Walf AA: **Effects of progesterone administration and APPswe+PSEN1Deltae9 mutation for cognitive performance of mid-aged mice.** *Neurobiology of learning and memory* 2008, **89:**17-26.
14. Thomas A, Burant A, Bui N, Graham D, Yuva-Paylor LA, Paylor R: **Marble burying reflects a repetitive and perseverative behavior more than novelty-induced anxiety.** *Psychopharmacology* 2009, **204:**361-373.
15. Young JW, Powell SB, Scott CN, Zhou X, Geyer MA: **The effect of reduced dopamine D4 receptor expression in the 5-choice continuous performance task: Separating response inhibition from premature responding.** *Behavioural brain research* 2011, **222:**183-192.
16. Dolan BM, Duron SG, Campbell DA, Vollrath B, Shankaranarayana Rao BS, Ko HY, Lin GG, Govindarajan A, Choi SY, Tonegawa S: **Rescue of fragile X syndrome phenotypes in Fmr1 KO mice by the small-molecule PAK inhibitor FRAX486.** *Proceedings of the National Academy of Sciences of the United States of America* 2013, **110:**5671-5676.

Additional File 1: Figure Legends

Additional File 1: Figure S1. Confirmation of Fragile X Protein Expression Knockout in the *Fmr1*/FVB Mouse Model.

The results of Western immunoblot analysis are illustrated for cerebral extracts from two knockout samples (FMR/FVB KO#1 and #2), two control samples (FVB WT#1 and #2), and one C57BL/6J sample.

Additional File 1: Figure S2. Acoustic Startle and Prepulse Inhibition.

(A) Fragile X knockout had decreased acoustic startle compared to FVB controls. Pulse intensities of 120dB produced a startle magnitude of 625 +/-65 in WT-Sal, and 657 +/-70 in WT-Sur animals, and 425 +/-58 in the KO-Sal, and 431 +/-59 in the KO-Sur animals. A pulse intensity of 105 dB in FVB controls produced a startle magnitude equivalent to 120 dB in the *Fmr1* knockout animals. **(B) Prepulse Inhibition Showed No Consistent Differences Between Fragile X Knockouts and Controls.** Significant differences in PPI were observed at different pulse intensities of 120 vs 105 dB. However, there was no difference between wild-type and KO genotypes at the same pulse intensities, and suramin did not alter this. 2-way ANOVA Prepulse intensity main effect $F(1,82) = 28.46$, $p < 0.0001$. Treatment Group $F(3,82) = 0.353$, p = ns. Suramin treatment did not change PPI. N = 10-12 per group, 16-week old males.

Additional File 1: Figure S3. Acyl-Carnitine Studies in *Fmr1* Knockout Mouse Models.

(A) Acyl-Carniitine Profile in the Fragile X Model on an FVB Background. (B) Plasma Acyl-Carnitines in the FVB Background are Lower than in C57BL/6J. (C) The Biochemical of Effect *Fmr1* Knockout on Absolute Acyl-Carnitine Concentrations is Similar in Both

FVB and C57BL6/J Genetic Backgrounds. (D) *Fmr1* Knockout on the C57BL/6 Background Does Not Produce Elevated Acyl-Carnitines.

Additional File 1: Figure S4. Western Blot Assay Linearity and Precision Analysis.

Linear regression analysis showed the assays to be linear with a mean correlation coefficient of r^2 = 0.984. **(A) pGSK3β. (B) StAR. (C) P2Y1. (D) P2X3. (E) Assay Precision.** SDS-PAGE and Western blots were prepared independently on 3 separate days using brain synaptosome samples from 5 animals from each of the two treatment groups (KO-Saline, KO-Suramin). Analysis of replicate results (N = 15 KO-Sal, 15 KO-Sur) revealed a mean assay precision of +/- 15%. **(F) 2-Way ANOVA Table of Western Blot Assays.** Analysis of variance revealed that the between-day assay variation contributed 0.3-1% of the variance. Suramin treatment explained 24-78% of the variance.

Additional File 1: Tables

Additional File 1: Table S1. Synaptic Proteins Interrogated and Antibodies Used.

Additional File 1: Table S2. Biochemical Pathways and Metabolites Interrogated.

Additional File 1: Table S3. Metabolites Changed by Antipurinergic Therapy in the Fragile X Model.

Additional File 1: Table S1. Synaptic Proteins Interrogated and Antibodies Used.

No.	Protein/Antibody Target	MW (KDa)	Response to Suramin KO-Sur/KO-Sal	Vendor	Primary Ab Dilution	Cat#
1	**PI3K**	**100**	**Down**	Cell Signaling	1,000	#3811
2	**Akt**	**60**	**Down**	Cell Signaling	1,000	#9272
3	**pGSK3β (Ser9)**	**50**	**Up**	Cell Signaling	1,000	#9323
4	**pS6K(Thr389)**	**70**	**Up**	Cell signaling	3,000	#9205
5	**APC**	**310**	**Down**	Cellsignaling	1,000	#2504
6	**P2Y1R**	**48**	**Up**	Alomone Labs	1,000	#APR-009
7	**P2X3R**	**44**	**Down**	Alomone Labs	1,000	#APR-026
8	**IP3R I**	**320**	**Up**	Cellsignaling	1,000	#3763
9	**GluR1**	**106**	**Down**	Abcam	1,000	#ab172971
10	**CB1**	**53**	**Down**	Abcam	1,000	#ab172970
11	**PPAR beta/delta**	**50**	**Up**	Abcam	1,000	#ab23673
12	**7-dehydrocholesterol reductase/7DHCR**	**54**	**Up**	Abcam	1,000	#ab103296
13	**Cholesterol 7 alpha-hydroxylase/CYP7A1**	**55**	**Up**	Abcam	1,000	#ab65596
14	**Steroidogenic acute regulatory protein/StAR**	**37**	**Up**	Cell Signaling	1,000	#8449
15	**C1qA**	**25**	**Down**	Abcam	1,000	#ab155052
16	**TAR DNA-binding protein 43/TDP43**	**45**	**Down**	Cell Signaling	1,000	#3449
17	**Amyloid β (Aβ) precursor protein/APP**	**100-140**	**Down**	Cellsignaling	1,000	#2452
18	pCAMKII(Thr286)	50, 60	None	Cellsignaling	1,000	#3361
19	pERK1/2(Thr202/Tyr204)	42, 44	None	Cell Signaling	10,000	#4370
20	pSTAT3(ser727)	86	None	Cell Signaling	1,000	#9134
21	P2Y2R	42	None	Alomone Labs	1,000	#APR-010
22	P2Y4R	41	None	Alomone Labs	1,000	#APR-006
23	P2X1R	45	None	Alomone Labs	1,000	#APR-022
24	P2X2R	44	None	Alomone Labs	1,000	#APR-025
25	P2X4R	43	None	Alomone Labs	1,000	#APR-024
26	P2X5R	47	None	Alomone Labs	1,000	#APR-005
27	P2X6R	50	None	Alomone Labs	1,000	#APR-013
28	P2X7R	68	None	Alomone Labs	1,000	#APR-004
29	Metabotropic glutamate receptor 5/mGluR5	132	None	Abcam	1,000	#ab76316
30	Nicotinic Acetylcholine Receptor alpha 7 /nAchR7α	50	None	Abcam	5,000	#ab23832
31	GABA A Receptor beta 3 /GABA-β3	54	None	Abcam	1,000	#ab4046
32	Dopamine Receptor D4/D4R	42	None	Alomone Labs	1,000	#ADR-004
33	ETFQO/ETFDH	65	None	Abcam	1,000	#ab126576
34	Methionine Sulfoxide Reductase A /MSRA	30	None	Abcam	1,000	#ab16803
35	Acetyl-CoA acetyltransferase 2/ACAT2	41	None	Cellsignal	1,000	#11814
36	HMGCoA Reductase/HMOCoAR	97	None	BioVision	500	#3952-100
37	Indoleamine 2,3-dioxygenase 1/IDO-1	45	None	Millipore	1,000	#MAB5412
38	p-mTOR(ser2448)	289	None	Cell Signaling	2,000	#2971
39	mTOR	289	None	Cell Signaling	2,000	#2972
40	pPERK(Thr980)	170	None	Cell Signaling	1,000	#3179
41	p-eIF2α(Ser51)	38	None	Cell Signaling	1,000	#9721
42	Nitro Tyrosine	10-200	None	Abcam	1,000	#ab7048
43	TGFβ Receptor I	50	None	Abcam	1,000	#ab31013
44	CB2	45	None	Abcam	1,000	ab45942
45	PGC1α	115	None	Abcam	1,000	#ab54481
46	PPARα	53	None	Santa Cruz	1,000	#sc-9000
47	CYP27A1	60	None	Abcam	1,000	#ab151987
48	pAkt(Thr308)	60	None	Cell Signaling	2,000	#4056
49	pAkt(Ser473)	60	None	Cell Signaling	2,000	#9018
50	PKC	82	None	Abcam	1,000	#ab19031
51	pPKC(Ser660)	80	None	Cell Signaling	1,000	#9371
52	nAchR beta2	70	None	Alomone Labs	1,000	#ANC-012
53	Postsynaptic Density protein 95/PSD95	95	None	Cell Signaling	4,000	#3450
54	Fragile X mental retardation protein/FMRP	80	None	Cell Signaling	2,000	#4317

Additional File 1: Table S2. Biochemical Pathways and Metabolites Interrogated.

No.	Pathway	Metabolites	No.	Pathway	Metabolites
1	1-Carbon, Folate, Formate, Glycine, Serine Metabolism	9	31	Pentose Phosphate, Gluconate Metabolism	11
2	Amino Acid Metabolism (not otherwise covered)	4	32	Phosphate and Pyrophosphate Metabolism	1
3	Amino-Sugar, Galactose, & Non-Glucose Metabolism	10	33	Phospholipid Metabolism	115
4	Bile Salt Metabolism	8	34	Phytanic, Branch, Odd Chain Fatty Acid Metabolism	1
5	Bioamines and Neurotransmitter Metabolism	11	35	Phytonutrients, Bioactive Botanical Metabolites	3
6	Biopterin, Neopterin, Molybdopterin Metabolism	2	36	Plasmalogen Metabolism	4
7	Biotin (Vitamin B7) Metabolism	1	37	Polyamine Metabolism	6
8	Branch Chain Amino Acid Metabolism	13	38	Purine Metabolism	41
9	Cardiolipin Metabolism	12	39	Pyrimidine Metabolism	31
10	Cholesterol, Cortisol, Non-Gonadal Steroid Metabolism	29	40	SAM, SAH, Methionine, Cysteine, Glutathione Metabolism	22
11	Eicosanoid and Resolvin Metabolism	36	41	Sphingolipid Metabolism	72
12	Endocannabinoid Metabolism	2	42	Taurine, Hypotaurine Metabolism	2
13	Fatty Acid Oxidation and Synthesis	39	43	Thyroxine Metabolism	1
14	Food Sources, Additives, Preservatives, Colorings, and Dyes	3	44	Triacylglycerol Metabolism	1
15	Forensic Drugs	1	45	Tryptophan, Kynurenine, Serotonin, Melatonin Metabolism	10
16	GABA, Glutamate, Arginine, Ornithine, Proline Metabolism	6	46	Tyrosine and Phenylalanine Metabolism	4
17	Gamma-Glutamyl and other Dipeptides	6	47	Ubiquinone and Dolichol Metabolism	4
18	Ganglioside Metabolism	12	48	Urea Cycle	4
19	Glycolysis and Gluconeogenesis Metabolism	18	49	Very Long Chain Fatty Acid Oxidation	3
20	Gonadal Steroids	2	50	Vitamin A (Retinol), Carotenoid Metabolism	3
21	Heme and Porphyrin Metabolism	4	51	Vitamin B1 (Thiamine) Metabolism	3
22	Histidine, Histamine, Carnosine Metabolism	5	52	Vitamin B12 (Cobalamin) Metabolism	3
23	Isoleucine, Valine, Threonine, or Methionine Metabolism	4	53	Vitamin B2 (Riboflavin) Metabolism	4
24	Ketone Body Metabolism	2	54	Vitamin B3 (Niacin, NAD+) Metabolism	8
25	Krebs Cycle	17	55	Vitamin B5 (Pantothenate, CoA) Metabolism	1
26	Lysine Metabolism	3	56	Vitamin B6 (Pyridoxine) Metabolism	5
27	Microbiome Metabolism	33	57	Vitamin C (Ascorbate) Metabolism	2
28	Nitric Oxide, Superoxide, Peroxide Metabolism	6	58	Vitamin D (Calciferol) Metabolism	2
29	OTC and Prescription Pharmaceutical Metabolism	3	59	Vitamin E (Tocopherol) Metabolism	1
30	Oxalate, Glyoxylate Metabolism	3	60	Vitamin K (Menaquinone) Metabolism	1
	Subtotal	304		Subtotal	369
	**TOTAL Pathways**	**60**		**TOTAL Metabolites**	**673**

Additional File 1: Table S3. Metabolites Changed by Antipurinergic Therapy in the Fragile X Model.

Metabolite	VIP Score	Metabolite	VIP Score
Xanthine	8.283	Myristoylcarnitine	1.8395
Hypoxanthine	6.9083	Trihexosylceramide 18:1/24:0	1.8222
Inosine	6.3985	Cholic acid	1.8062
LTB4	4.7929	Octanoylcarnitine	1.7888
Guanosine	4.1962	Pimelylcarnitine	1.7778
1-Methylnicotinamide	3.4567	Ceramide (d18:1/26:0)	1.7619
11-Dehydro-thromboxane B2	3.0285	PG(16:0/16:0)	1.7575
4-hydroxyphenyllactic acid	2.9524	Dodecenoylcarnitine	1.7435
L-cystine	2.8156	Nicotinamide N-oxide	1.724
Hexanoylcarnitine	2.766	Dodecanoylcarnitine	1.6983
Dihexosylceramide (18:1/24:1)	2.7087	L-Homocysteic acid	1.6739
Ceramide (d18:1/24:1)	2.6984	9-Decenoylcarnitine	1.6702
Ceramide (d18:1/24:0 OH)	2.6743	Hydroxyisocaproic acid	1.6696
2,3-Diphosphoglyceric acid	2.6413	Propionic acid	1.6633
PI (26:1)	2.5143	5-alpha-Cholestanol	1.6542
Dihexosylceramide (18:1/20:0)	2.5094	Glyceric acid 1,3-biphosphate	1.6112
Ceramide (d18:1/16:0 OH)	2.4973	Bismonoacylphospholipid (18:1/18:0)	1.6108
Trihexosylceramide 18:1/16:0	2.2984	3-methylphenylacetic acid	1.6055
Cysteineglutathione disulfide	2.2284	Cytidine	1.5738
dTDP-D-glucose	2.1762	Oxaloacetic acid	1.5682
Trihexosylceramide 18:1/22:0	2.1755	9-Hexadecenoylcarnitine	1.5637
Bismonoacylphospholipid (18:1/18:1)	2.0984	Dehydroisoandrosterone 3-sulfate	1.5627
Malondialdehyde	2.0928	Ceramide (d18:1/20:1)	1.5607
PC (18:0/20:3)	2.087	11(R)-HETE	1.5384
3, 5-Tetradecadiencarnitine	2.0594	PE (38:5)	1.5338
14,15-epoxy-5,8,11-eicosatrienoic acid	1.9964	Pyridoxamine	1.5335
Cardiolipin (24:1/24:1/24:1/14:1)	1.9754	11,12-DiHETrE	1.5284
Trihexosylceramide 18:1/24:1	1.9105	Sedoheptulose 7-phosphate	1.5159
8,9-Epoxyeicosatrienoic acid	1.8643	AICAR	1.5150

Additional File 1: Figure S1

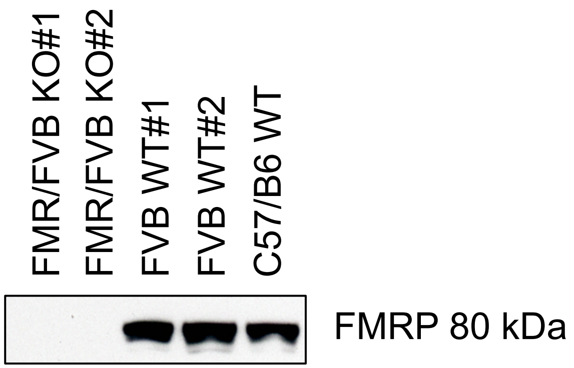

Naviaux, *et al.*, 2015

Additional File 1: Figure S2

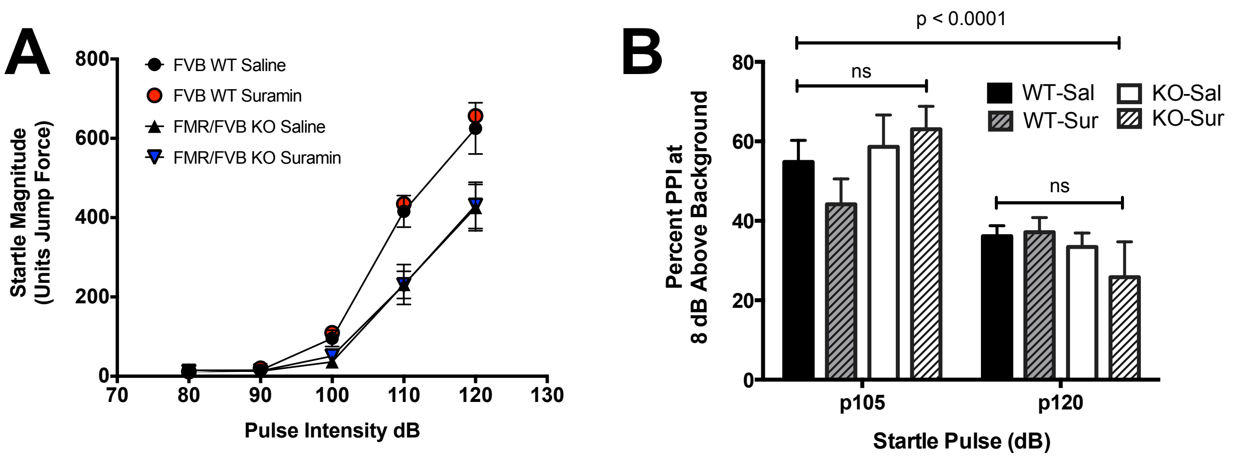

Naviaux, *et al.*, 2015

Additional File 1: Figure S3A

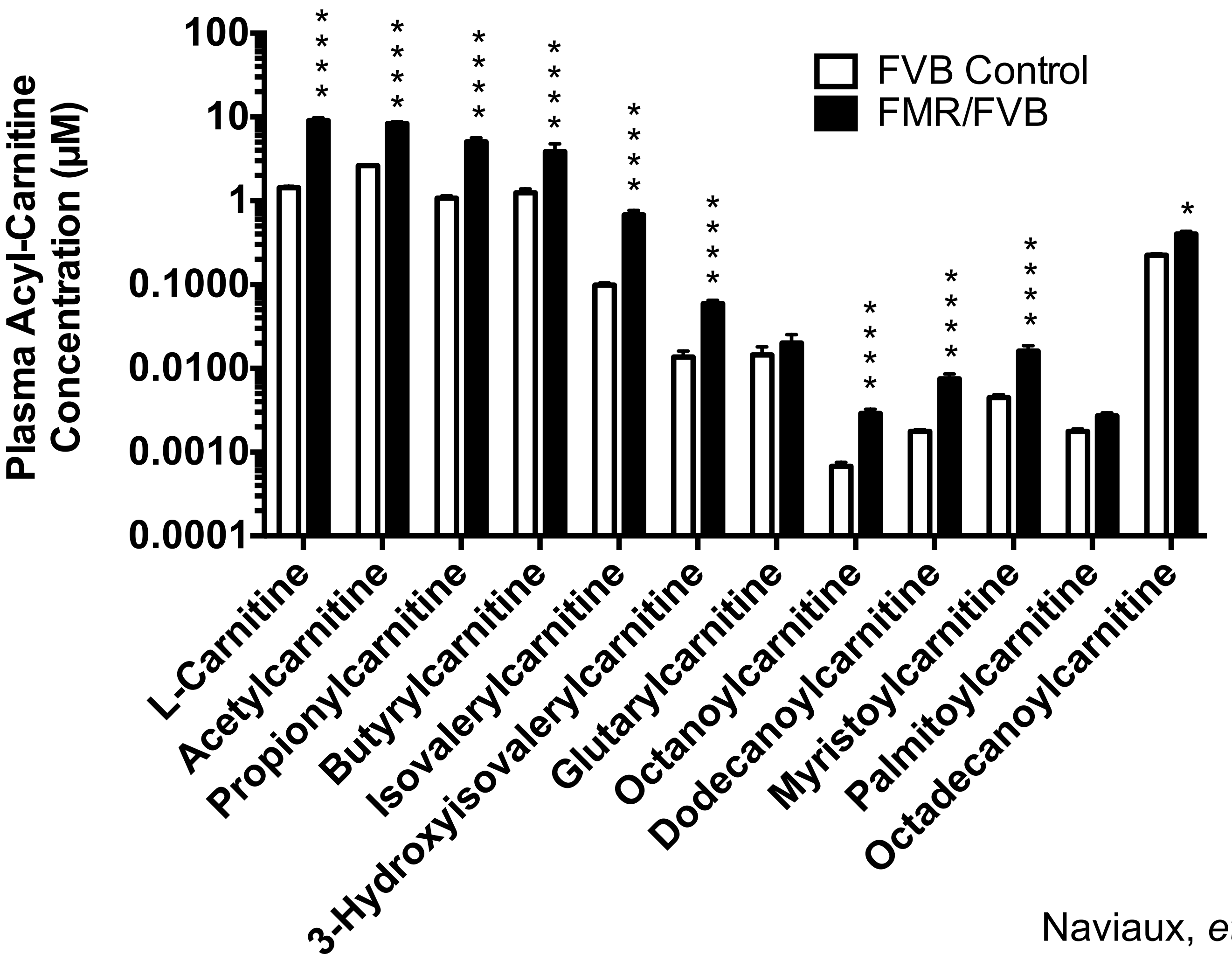

Naviaux, *et al.*, 2015

Additional File 1: Figure S3B

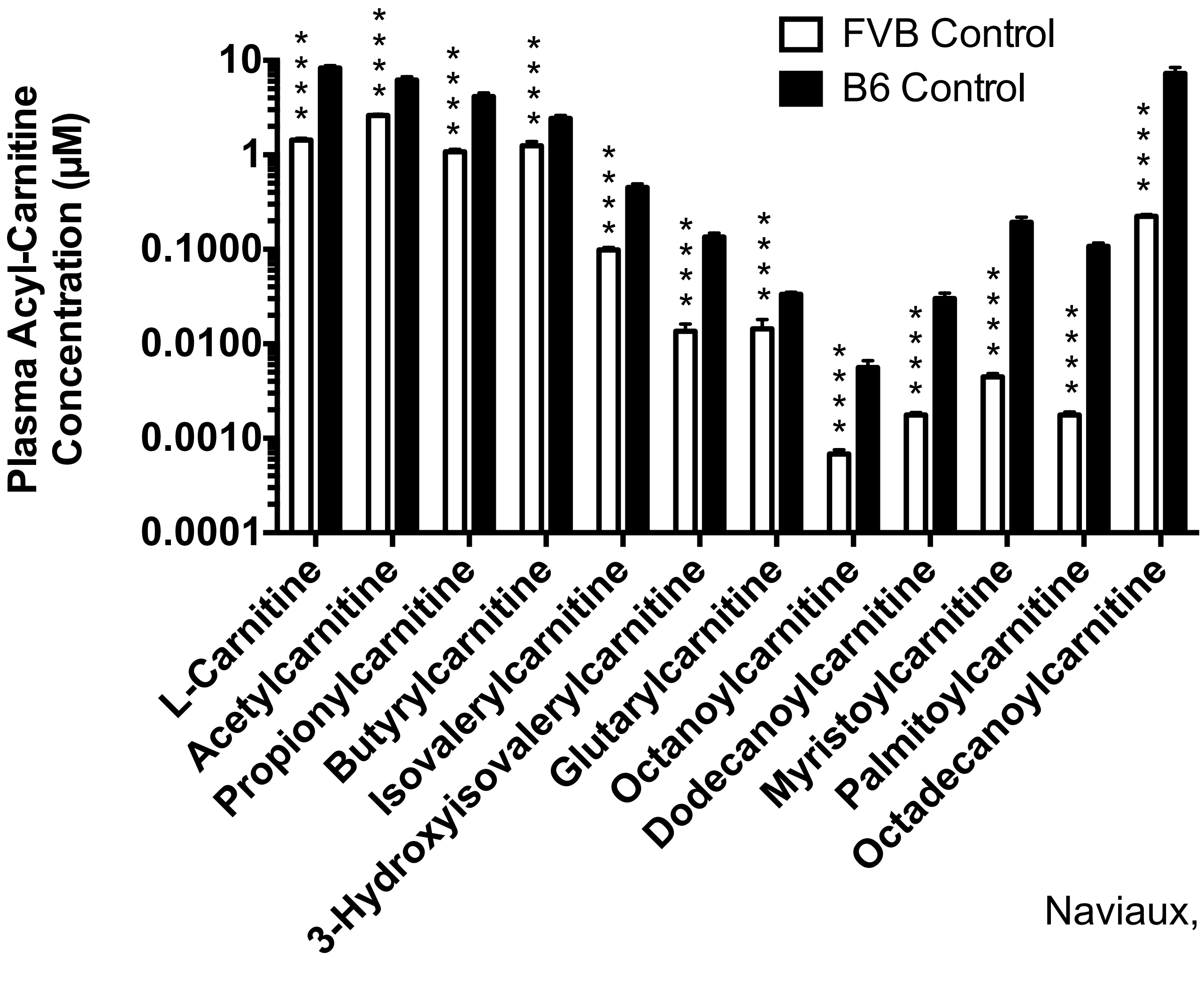

Naviaux, *et al.*, 2015

Additional File 1: Figure S3C

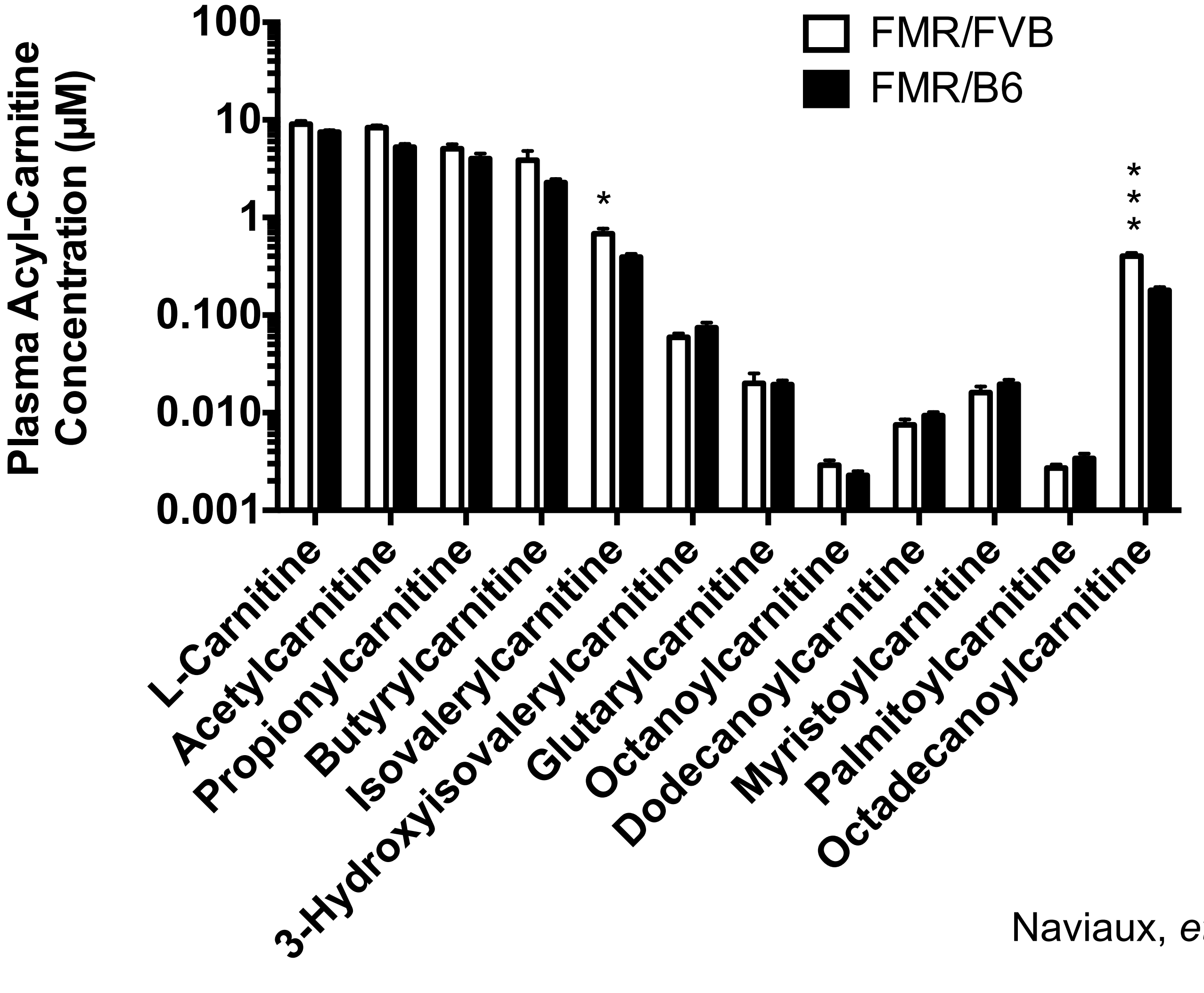

Naviaux, *et al.*, 2015

Additional File 1: Figure S3D

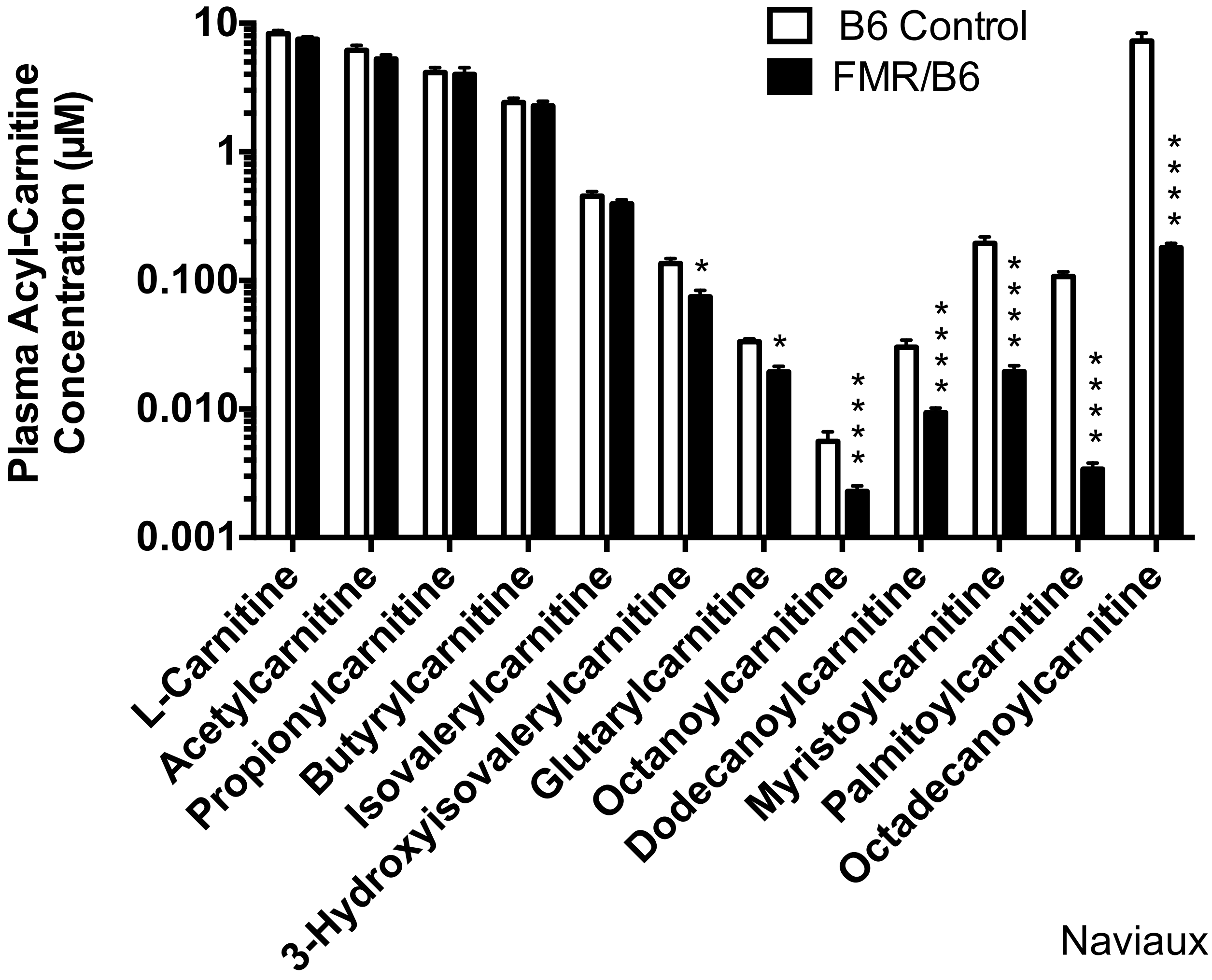

Naviaux, *et al.*, 2015

Additional File 1: Figure S4

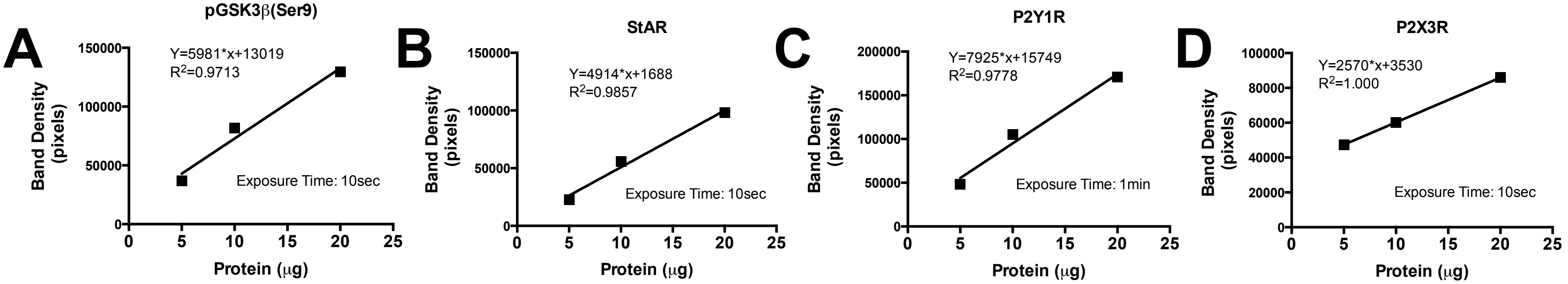

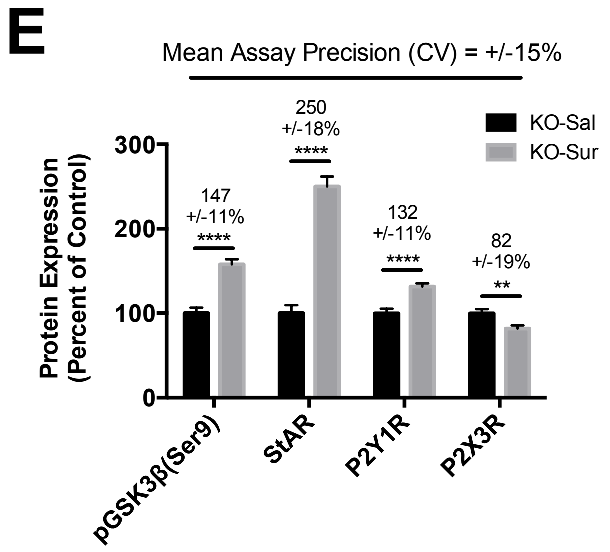

F

Protein	Samples/ Group	Replicates (Days)	Total Samples/ Group	Groups (KO-Sal v KO-Sur)	Percent of Total Variation: Day x Treatment Interaction	Percent of Total Variation: Day	Percent of Total Variation: Suramin Treatment	F	P value
pGSK	5	3	15	2	0.3%	0.3%	66.1%	(1,24) = 47.7	<0.0001
StAR	5	3	15	2	1%	1%	77.9%	(1,24) = 93.4	<0.0001
P2Y1R	5	3	15	2	0.6%	0.6%	45.5%	(1,24) = 20.5	<0.0001
P2X3R	5	3	15	2	0.4%	0.4%	23.6%	(1,24) = 7.5	<0.01

Naviaux, *et al.*, 2015
